# Integrated strategies in meniscus tissue engineering: from biomaterials to stem cell–driven regeneration

**DOI:** 10.3389/fbioe.2026.1691953

**Published:** 2026-03-03

**Authors:** Puzhen Song, Hongguang Chen, Hebin Ma, Yuanbo Zhou, Yadong Zhang

**Affiliations:** 1 Senior Department of Orthopedics, Chinese PLA Hospital, Beijing, China; 2 Senior Department of Orthopedics, the Fourth Medical Center of PLA General Hospital, Beijing, China

**Keywords:** 3D/4D printing, chondrocytes, meniscus, meniscus tissue engineering, stem cells (MSCs)

## Abstract

The meniscus is a fibrocartilaginous tissue essential for load distribution, shock absorption, and knee joint stability, yet its intrinsic healing potential is limited, particularly in the avascular inner zone. Conventional treatments such as partial meniscectomy, repair, or transplantation often fail to restore long-term biomechanical and biological function, underscoring the need for regenerative strategies. Meniscus tissue engineering (TE) has emerged as a promising approach that combines biomaterial scaffolds with stem cells to recreate the structural and functional complexity of the native tissue. This narrative review summarizes recent advances in scaffold design and cell-based therapies for meniscus repair. Natural materials such as collagen, alginate, and silk fibroin provide biocompatibility and bioactivity but lack sufficient mechanical strength, whereas synthetic polymers including PGA, PLA, PLGA, and polyurethane offer tunable degradation and structural reinforcement but are biologically inert. Composite scaffolds that integrate these material classes—through multiphase, gradient, or layered designs—represent a promising strategy to replicate zonal heterogeneity and anisotropic mechanics. On the cellular side, bone marrow–, adipose-, and synovium-derived mesenchymal stem cells have demonstrated potential for zone-specific regeneration, while induced pluripotent stem cells present opportunities for patient-specific therapies but remain limited by safety concerns. Advances in cell seeding strategies, including dynamic perfusion and 3D bioprinting, have further improved scaffold–cell integration. Finally, emerging technologies such as 3D/4D printing, smart responsive biomaterials, controlled drug delivery, dynamic bioreactors, and AI-assisted scaffold design provide new opportunities to overcome persistent challenges of vascularization, mechanical anisotropy, and clinical translation. While significant obstacles remain, the convergence of materials science, stem cell biology, advanced fabrication, and computational modeling offers a promising roadmap toward clinically viable meniscus regeneration.

## 1 Introduction

### 1.1 Clinical background: epidemiology and challenges of meniscal injury

The meniscus is a fibrocartilaginous structure of the knee joint that plays a crucial role in load distribution, shock absorption, joint stabilization, and lubrication. Meniscal injuries are among the most common musculoskeletal disorders, with an estimated prevalence of 12%–14% in the general population and up to 30%–40% in athletes (Makris et al., 2011; Papalia et al., 2013). The incidence is expected to rise further due to increased sports participation and population aging. Meniscal tears not only cause pain and functional impairment but also predispose patients to progressive cartilage degeneration and osteoarthritis (OA), ultimately increasing the likelihood of total knee arthroplasty. The intrinsic healing potential of the meniscus is extremely limited, particularly in the avascular inner two-thirds (white zone), which lacks the vascular supply necessary for effective tissue repair. This limited regenerative capacity renders meniscal injury a persistent clinical challenge (Mcnulty and Guilak, 2015; Englund et al., 2003).

From a clinical perspective, treatment decisions for meniscal tears are strongly influenced by patient-related and lesion-specific factors. Patient age remains one of the most deterministic variables, as younger individuals with traumatic tears generally exhibit higher healing potential and are more suitable for meniscal preservation strategies, whereas older patients more frequently present with degenerative tears associated with early joint degeneration (Doral et al., 2018; Beaufils et al., 2017a).

In addition to age, symptom patterns play a critical role in guiding treatment. Repetitive mechanical locking, catching, or instability often indicates an unstable tear pattern and favors surgical intervention over prolonged conservative management. In contrast, stable degenerative tears without mechanical symptoms are increasingly regarded as part of a broader osteoarthritic process rather than isolated mechanical lesions, and may benefit more from non-operative or biologically supportive strategies (Beaufils et al., 2017b; Beaufils and Pujol, 2017; Siemieniuk et al., 2017).

These evolving clinical perspectives underscore the need to align emerging regenerative approaches, including tissue engineering, with clearly defined patient populations and tear characteristics rather than applying a uniform treatment paradigm (Beaufils et al., 2017b).

### 1.2 Limitations of current treatments: meniscectomy and transplantation

Conventional treatment strategies for meniscal injury include partial meniscectomy, meniscal repair, and meniscal transplantation using allografts or synthetic implants (Vrancken et al., 2013). Partial meniscectomy may provide short-term symptomatic relief; however, removal of meniscal tissue disrupts load transmission and increases tibiofemoral contact stress, thereby accelerating cartilage degeneration and the progression of osteoarthritis (Papalia et al., 2011). Meniscal repair is generally preferred for tears located in the vascularized outer (red) zone, particularly in younger patients, but its success rate declines markedly in the avascular white zone and in complex or degenerative tear patterns (Bansal et al., 2021).

Meniscal allograft transplantation is commonly employed in patients with subtotal or total meniscectomy, yet its clinical application is constrained by several inherent limitations (Lee et al., 2012; Figueroa et al., 2019). These include restricted graft availability, challenges in accurate size matching, variable biological integration, and concerns related to immunogenicity and disease transmission, despite rigorous screening procedures (Samitier et al., 2015). Synthetic meniscal implants have also been developed as alternatives; however, most currently available designs lack sufficient mechanical durability and fail to provide the biological cues necessary for long-term functional restoration. Collectively, these limitations underscore the inability of existing treatments to fully replicate the complex biomechanical and biological functions of the native meniscus (Barber, 2018; Longo et al., 2013).

**FIGURE 1:**
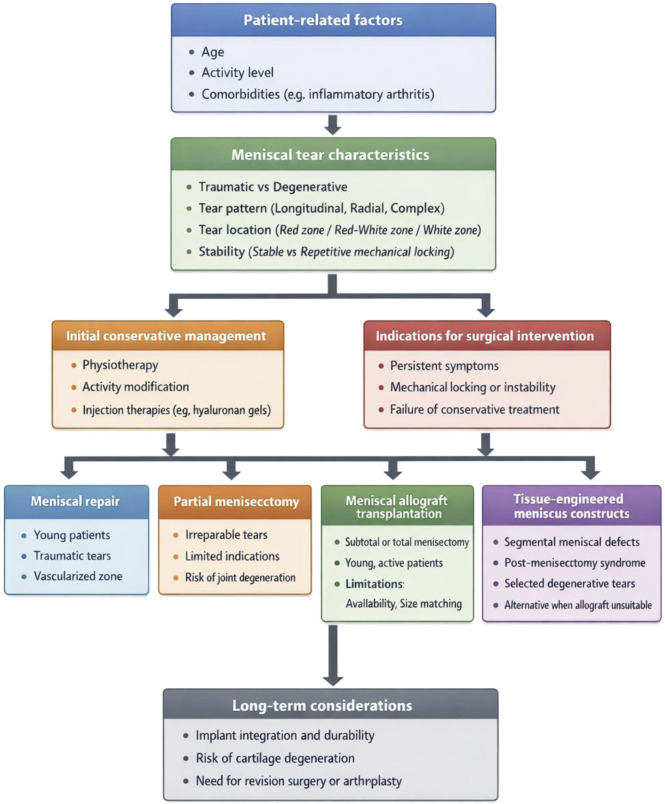
This schematic illustrates a stepwise clinical decision-making algorithm for the management of meniscal tears. Treatment selection is guided by patient-related factors (such as age, activity level, and comorbidities), tear characteristics (including etiology, pattern, location, and stability), and symptom severity. Conservative management remains the first-line approach for selected patients, whereas surgical intervention is indicated in the presence of persistent symptoms or mechanical instability. Conventional surgical options include meniscal repair, partial meniscectomy, and meniscal allograft transplantation. Tissue-engineered meniscus constructs are positioned as emerging therapeutic alternatives for specific indications, such as segmental meniscal defects and post-meniscectomy syndrome, particularly when allograft transplantation is unsuitable. Long-term considerations include implant integration, durability, and the risk of progressive joint degeneration.

To better contextualize emerging regenerative strategies within routine clinical practice, a stepwise treatment algorithm for meniscal tears is presented in Figure 1. This schematic integrates patient age, tear etiology, symptom severity, and joint condition to illustrate the progression from conservative management to surgical intervention. Within this framework, tissue-engineered meniscus constructs are positioned as potential solutions for selected indications, including segmental meniscal defects and post-meniscectomy syndrome, particularly in patients who are unsuitable candidates for allograft transplantation. Importantly, tissue engineering is not proposed as a universal replacement for conventional treatments, but rather as a targeted approach to address specific unmet clinical needs (Wang H. et al., 2025).

Compared with allograft transplantation, tissue-engineered meniscus constructs offer theoretical advantages such as customizable geometry, controlled material composition, and the potential for enhanced biological integration. Nevertheless, these approaches remain limited by a lack of long-term clinical evidence, as well as unresolved challenges related to mechanical durability, manufacturing standardization, and regulatory approval (Klarmann et al., 2021; Van Minnen and Van Tienen, 2024). A systematic comparison between meniscal allograft transplantation and tissue-engineered meniscus constructs is provided in Table 1.

**TABLE 1:** Comparison between meniscal allograft transplantation and tissue-engineered meniscus constructs.

Aspect	Meniscal allograft transplantation	Tissue-engineered meniscus constructs
Clinical maturity	Established clinical procedure with decades of use	Emerging approach, largely in preclinical or early clinical stages
Availability	Limited by donor supply and tissue banking	Potentially scalable and customizable
Size matching	Size mismatch remains a common challenge	Geometry can be patient-specific and customizable
Biological integration	Variable integration with host tissue	Theoretical potential for enhanced integration depending on scaffold design and cell source
Immunogenicity	Low but present; risk of immune response	Low for acellular scaffolds; cell-based constructs may introduce immunological concerns
Disease transmission risk	Present despite rigorous screening	Minimal, depending on material and cell source
Mechanical properties	Native tissue properties initially preserved	Highly dependent on scaffold material and architecture
Long-term durability	Reported graft degeneration and extrusion in some cases	Long-term mechanical performance remains largely unknown
Surgical complexity	Technically demanding, requires precise fixation	Variable; may be simplified depending on construct design
Regulatory status	Approved and clinically available in many regions	Mostly investigational; regulatory approval pending
Clinical evidence	Supported by mid- to long-term clinical outcome studies	Limited clinical data; predominantly experimental
Typical indications	Young, active patients after subtotal or total meniscectomy	Segmental defects, post-meniscectomy syndrome, or cases unsuitable for allograft
Key limitations	Graft availability, size mismatch, variable outcomes	Mechanical durability, standardization, regulatory and translational challenges

Beyond surgical interventions, reparative hyaluronan-based gels have gained increasing attention as minimally invasive or adjunctive treatment options for selected meniscal injuries (Zorzi et al., 2015). These injectable formulations may provide temporary biomechanical support, modulate intra-articular inflammation, and improve the joint microenvironment, particularly in early-stage or degenerative meniscal lesions (Berton et al., 2020). Although hyaluronan gels do not restore native meniscal architecture, their growing clinical use highlights the importance of biologically supportive strategies that may complement or delay surgical intervention and inform the design of injectable or hydrogel-based tissue-engineered therapies (Wang G. et al., 2025).

Meniscal tears may also occur in the context of inflammatory joint diseases, such as rheumatoid arthritis, where the underlying pathophysiology differs substantially from purely mechanical injury. In these patients, chronic synovitis, altered immune responses, and the use of immunosuppressive medications may impair tissue healing and influence surgical outcomes (Meng et al., 2018; Kwon et al., 2019). Consequently, treatment strategies for meniscal lesions in inflammatory joints require careful consideration, and the applicability of regenerative or tissue-engineered approaches remains uncertain. Elucidating the interactions between engineered constructs and inflammatory microenvironments will be critical for extending these technologies to broader patient populations (George and Baker, 2019).

### 1.3 Importance of tissue engineering: scaffold and stem cell strategies

**FIGURE 2:**
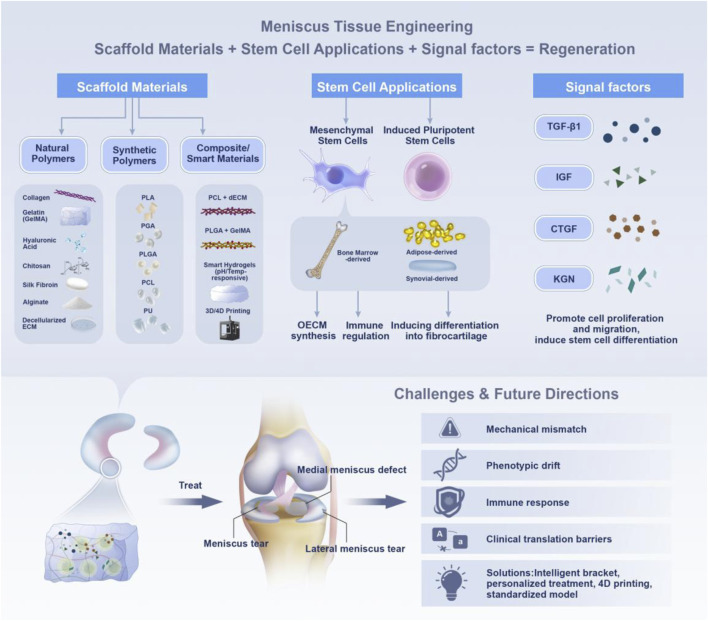
This diagram illustrates the key components and strategies involved in meniscus tissue engineering. It emphasizes the combination of scaffold materials (both natural and synthetic polymers, as well as composite/smart materials), stem cell applications (including mesenchymal stem cells and induced pluripotent stem cells), and signaling factors crucial for regenerating functional meniscal tissue. The diagram also highlights the challenges of mechanical mismatch, phenotypic drift, immune response, and clinical translation, proposing solutions like personalized treatments and 3D/4D printing technologies.

Tissue engineering (TE) has emerged as a promising paradigm to overcome the shortcomings of current treatments by combining biomaterial scaffolds, seed cells, and bioactive factors to regenerate functional meniscal tissue (Niu et al., 2016). An ideal scaffold should mimic the extracellular matrix (ECM) environment of the meniscus, providing mechanical support, biocompatibility, and a controlled degradation profile while facilitating cell adhesion, proliferation, and differentiation (Stocco T. D. et al., 2022 Shimomura et al., 2018). Various natural and synthetic biomaterials have been investigated for scaffold fabrication, including collagen, silk fibroin, polylactic acid (PLA), and polycaprolactone (PCL), with ongoing efforts to optimize composite structures that integrate the advantages of multiple material classes. Stem cells, particularly mesenchymal stem cells (MSCs) derived from bone marrow, adipose tissue, or synovium, have demonstrated the potential to differentiate toward fibrochondrocyte-like phenotypes and contribute to matrix regeneration. Induced pluripotent stem cells (iPSCs) have also been explored, although their long-term stability and safety require further validation. Importantly, the integration of advanced technologies—such as 3D and 4D bioprinting, dynamic bioreactors, and intelligent drug delivery systems—offers new opportunities to fabricate biomimetic scaffolds and regulate cell fate, bringing meniscus tissue engineering closer to clinical translation (Makris et al., 2011; Zellner et al., 2010; Baysan et al., 2022) (Figure 2).

This is a narrative review that highlights representative advances and emerging trends in meniscus tissue engineering. Specifically, we summarize natural and synthetic scaffolds as well as composite designs applied to meniscus regeneration, stem cell sources and their therapeutic potential, and emerging technologies including 3D/4D printing, responsive biomaterials, and artificial intelligence (AI)-assisted scaffold design. Our goal is to provide readers with a structured overview of the field while emphasizing meniscus-specific engineering considerations and outstanding challenges that must be addressed to achieve durable clinical outcomes (Mcnulty and Guilak, 2015; Zhang et al., 2018; Zellner et al., 2013).

## 2 Meniscus structure and composition

### 2.1 Anatomical zones: red zone, white zone, and red–white junction

The meniscus is a semilunar fibrocartilaginous tissue situated between the femoral condyles and tibial plateau, consisting of medial and lateral components. Each meniscus displays distinct morphological features: the medial meniscus is C-shaped and more firmly attached to the tibial plateau, whereas the lateral meniscus is more circular and exhibits greater mobility. For both structures, the internal architecture is highly heterogeneous, and its function is determined largely by its zonal anatomy (Makris et al., 2011; Fox et al., 2012).

Traditionally, the meniscus is divided into three zones based on vascularization: the outer third or red zone, the inner third or white zone, and the intermediate red–white zone (Arnoczky and Warren, 1982). The red zone, being adjacent to the synovial capsule, is well vascularized and demonstrates a higher healing potential following injury. In contrast, the white zone is avascular, relying solely on diffusion from synovial fluid for nutrient supply, and is consequently characterized by minimal intrinsic regenerative capacity. The red–white junction possesses an intermediate level of vascularization and represents a transition between the two extremes. This zonal organization not only dictates the biological behavior of cells but also has significant clinical implications, as meniscal tears located in the vascularized zone are more amenable to surgical repair (Cipolla et al., 1992).

### 2.2 Vascular supply and metabolic gradients

Meniscal vascularization is established during early development but regresses significantly with age. In neonates, the entire meniscus is vascularized; however, by the age of 10, only the peripheral rim retains vascularity, and in adults, less than 25%–30% of the meniscal tissue is directly vascularized. Blood supply arises primarily from branches of the medial and lateral genicular arteries, forming a perimeniscal capillary plexus that penetrates the outer meniscal rim (Orellana et al., 2024).

This vascular gradient creates corresponding differences in metabolic activity. Cells in the red zone exhibit higher proliferation rates and metabolic flexibility, whereas cells in the white zone rely largely on anaerobic metabolism due to limited nutrient diffusion. Consequently, healing is predominantly restricted to the vascularized peripheral region, and tears in the avascular white zone rarely heal spontaneously. These characteristics highlight the importance of designing scaffolds and cell-based strategies capable of addressing the nutritional and metabolic challenges posed by the avascular zone (Makris et al., 2011; Fox et al., 2015).

### 2.3 Cellular phenotypes: fibrochondrocytes versus fibroblast-like cells

**FIGURE 3:**
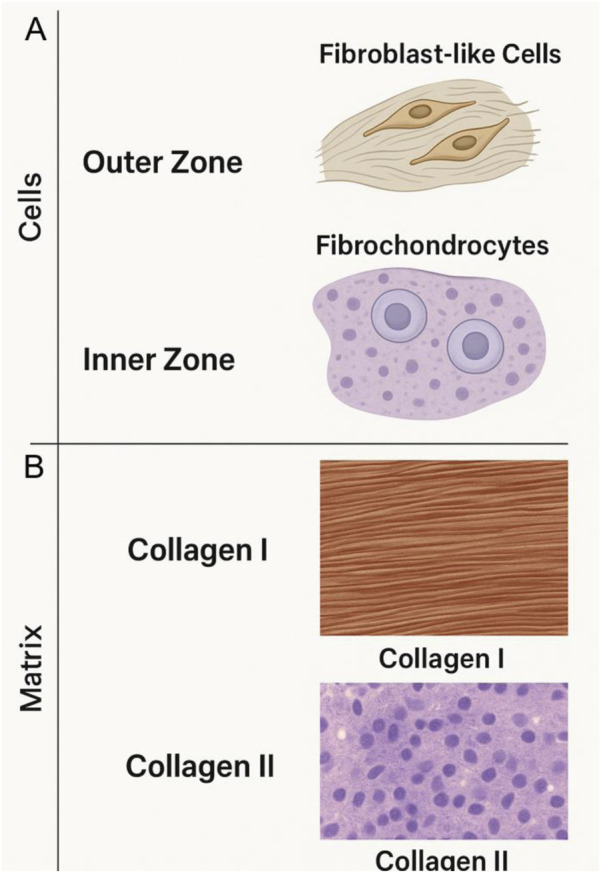
This diagram compares the distinct cellular phenotypes of the outer red zone **(A)** and inner white zone **(B)** of the meniscus, focusing on fibroblast-like cells and fibrochondrocytes. Fibroblast-like cells, found in the vascularized red zone, predominantly produce type I collagen, contributing to the meniscus’s tensile strength. In contrast, fibrochondrocytes in the inner white zone, which is avascular, produce a proteoglycan-rich matrix containing type I and type II collagen and glycosaminoglycans (GAGs), important for compressive resistance. The figure further emphasizes the role of extracellular matrix (ECM) components, such as collagen and proteoglycans, in the mechanical resilience and functionality of the meniscus tissue.

The meniscus harbors a heterogeneous population of cells that differ by anatomical zone. Two principal phenotypes have been widely described in the literature: fibroblast-like cells and fibrochondrocytes (Hellio Le Graverand et al., 2001) (Figure 3). Fibroblast-like cells, located in the vascularized outer red zone, exhibit elongated morphologies and predominantly synthesize type I collagen, thereby contributing to the tensile properties of the tissue (Lee et al., 2025). In contrast, fibrochondrocytes in the avascular inner white zone display a rounded morphology and produce a proteoglycan-rich matrix containing both type I and type II collagen as well as glycosaminoglycans (GAGs) (Sun et al., 2020). Some investigations have also reported transitional phenotypes in the red–white zone, indicating a spectrum rather than strictly binary classification (Mcdevitt and Webber, 1990; Petersen and Tillmann, 1998).

This cellular heterogeneity has important implications for tissue engineering. For instance, scaffolds designed for the outer zone may require fibrogenic cues to support fibroblast-like phenotypes, whereas inner-zone regeneration demands environments promoting chondrogenic differentiation (Son et al., 2013). Recent studies have highlighted that stem cells such as synovial MSCs or BMSCs can be directed toward either phenotype depending on scaffold composition and mechanical loading. Such insights emphasize the need to tailor biomaterial and cell strategies to the meniscus’s zonal biology (Baker et al., 2009).

### 2.4 Extracellular matrix composition: collagens, proteoglycans, and glycosaminoglycans

The extracellular matrix (ECM) of the meniscus is highly specialized and confers its mechanical resilience. Collagen is the most abundant component, accounting for approximately 60%–70% of the dry weight. Type I collagen dominates the outer vascularized region, providing tensile strength and resistance to stretching. Type II collagen is enriched in the inner region, where it contributes to compressive resistance, although its proportion remains lower than in articular cartilage. Minor collagens such as type III, V, and VI have also been identified, playing roles in fibril organization and matrix stability (Cheung, 1987).

Proteoglycans, though accounting for only 1%–2% of the wet weight, are crucial for resisting compressive loads. Aggrecan is the most abundant proteoglycan, forming aggregates with hyaluronic acid and retaining water to generate osmotic pressure. Other proteoglycans, such as decorin, biglycan, and fibromodulin, regulate collagen fibrillogenesis and matrix assembly (Fuhrmann et al., 2015).

Glycosaminoglycans (GAGs), including chondroitin sulfate and keratan sulfate, are unevenly distributed across the meniscus, with higher concentrations in the inner white zone. This regional variation corresponds to the zone’s requirement for compressive strength, contrasting with the outer zone’s emphasis on tensile resistance (Hellio Le Graverand et al., 2001; Bilgen et al., 2018).

### 2.5 Fiber orientation and biomechanical roles: circumferential bundles and tie fibers

The functional behavior of the meniscus is dictated not only by its biochemical composition but also by the intricate architecture of its collagen network. The predominant structural motif is the circumferential collagen fiber bundle, aligned parallel to the peripheral rim. This orientation allows the meniscus to resist hoop stresses generated during load-bearing activities. When axial compressive forces are applied, they are transmitted as tensile forces along the circumferential fibers, preventing meniscal extrusion (Fithian et al., 1990).

In addition to circumferential fibers, radial tie fibers traverse the tissue perpendicularly, anchoring circumferential bundles and preventing longitudinal splitting. A superficial mesh of randomly oriented fibers covers the meniscal surface, contributing to shear resistance. This hierarchical organization ensures that the meniscus withstands complex multiaxial forces encountered during joint motion. Reconstructing these structural features remains one of the greatest challenges in meniscus tissue engineering, as anisotropy is essential for restoring native mechanical function (Makris et al., 2011; Hellio Le Graverand et al., 2001; Andrews et al., 2013).

### 2.6 Functional implications: load bearing, shock absorption, and joint stability

The unique combination of zonal anatomy, vascularization, cellular heterogeneity, and ECM architecture enables the meniscus to fulfill multiple biomechanical roles. By increasing the contact area between the femoral condyles and tibial plateau, the meniscus reduces peak contact stresses by up to 50%–70%. Its viscoelastic properties allow it to absorb shock and dissipate energy during dynamic activities such as walking and jumping. Moreover, the circumferential collagen network stabilizes the knee by constraining excessive tibial translation and rotation (Makris et al., 2011; Ahmed and Burke, 1983).

Loss of meniscal integrity disrupts these functions, leading to joint instability, abnormal stress concentration, cartilage degeneration, and ultimately OA. Thus, any tissue engineering approach must not only restore cellular and biochemical composition but also reproduce the complex hierarchical organization necessary for biomechanical performance (Makris et al., 2011; Englund et al., 2003; Stein et al., 2010; Huey et al., 2012).

The meniscus is a highly specialized fibrocartilaginous tissue with distinct zonal anatomy, vascular gradients, heterogeneous cell populations, and a complex ECM architecture tailored for both tensile and compressive loading. These features are directly linked to its critical role in load transmission, shock absorption, and joint stability. Understanding this intricate structure is essential for guiding scaffold design, selecting appropriate cell sources, and identifying biological and biomechanical targets in meniscus tissue engineering (Stocco E. et al., 2022; Vasiliadis et al., 2021; Vo et al., 2012).

## 3 Engineering considerations for meniscus tissue engineering

Beyond cellular composition, biomimetic replication of the native meniscal architecture is essential for functional restoration. The highly organized circumferential collagen fiber network and radial tie fibers enable the meniscus to withstand complex multiaxial loading.

Therefore, tissue-engineered meniscus constructs must be designed not only to support appropriate cell phenotypes, but also to reproduce native fiber orientation and anisotropic mechanical behavior. Failure to replicate this hierarchical structure may compromise load transmission and long-term durability, regardless of cellular viability.

### 3.1 Vascularization limitations

One of the most fundamental challenges in meniscus tissue engineering lies in replicating the intrinsic vascular gradient. Unlike articular cartilage, which is completely avascular, the meniscus contains both vascularized and avascular regions. The peripheral red zone benefits from a capillary plexus supplied by the genicular arteries, whereas the inner white zone is entirely dependent on diffusion from synovial fluid. This heterogeneity creates significant obstacles for regeneration (Seol et al., 2017).

Scaffolds must be designed not only to support cellular infiltration but also to promote nutrient transport and, ideally, induce neo-vascularization in otherwise poorly perfused regions. Attempts to incorporate angiogenic factors such as vascular endothelial growth factor (VEGF) or to co-culture endothelial cells with stem cells have demonstrated some promise, yet excessive vascularization of the inner meniscus may disrupt its native physiology. Thus, unlike cartilage engineering—where a uniformly avascular environment is replicated—meniscus engineering requires scaffolds with spatially controlled vascular cues to recapitulate its zonal biology. Achieving this delicate balance remains a major hurdle to clinical translation (Grogan et al., 2020).

### 3.2 Circumferential structure and tensile loading requirements

The anisotropic architecture of the meniscus is unique among fibrocartilaginous tissues. Circumferential collagen bundles aligned along the peripheral rim resist hoop stresses, while radial tie fibers prevent longitudinal splitting. Replicating this alignment is critical for restoring function. Traditional scaffolds often lack directional fiber organization, resulting in isotropic mechanical properties that fail to mimic native anisotropy (Petersen and Tillmann, 1998; Cui and Min, 2007).

Advanced techniques such as electrospinning, aligned 3D printing, and fiber-reinforced composites have been employed to impose directionality within scaffolds. For instance, scaffolds with circumferentially oriented fibers demonstrate superior tensile strength and better integration with host tissue compared with randomly oriented constructs. This requirement sets meniscus tissue engineering apart from cartilage engineering, where isotropic scaffolds can adequately replicate compressive properties. In the meniscus, failure to restore the circumferential structure may result in inadequate hoop stress resistance, extrusion of the implant, and accelerated degeneration of the articular cartilage (Baker et al., 2011; Spalazzi et al., 2006).

### 3.3 Shock absorption and long-term durability

The meniscus functions as both a load distributor and a shock absorber. During gait and athletic activities, it experiences a combination of compressive, tensile, and shear forces, often at magnitudes exceeding several times body weight. Replicating such multifunctional mechanical behavior is exceedingly complex. A scaffold must provide immediate load-bearing capacity to prevent joint overloading while also permitting remodeling and integration with host tissue (Wang et al., 2012).

Biodegradable scaffolds face a trade-off: rapid degradation undermines mechanical stability, while prolonged persistence may hinder matrix deposition and remodeling. Moreover, the viscoelastic properties that allow the meniscus to dissipate energy and recover its shape after deformation are difficult to reproduce in synthetic constructs. While cartilage engineering focuses largely on compressive stiffness and GAG content, meniscus engineering must integrate tensile strength, shear resistance, and viscoelastic recovery into a single construct. Long-term durability under repeated cyclic loading is another critical consideration, as implants must withstand millions of gait cycles per year without failure (Sweigart and Athanasiou, 2005).

### 3.4 Surgical implantation and fixation issues

Even when promising scaffolds are developed *in vitro*, their clinical translation depends on practical considerations related to surgical implantation. The meniscus is anchored to the tibial plateau at its anterior and posterior horns, with peripheral attachments to the joint capsule and ligaments. Any implant must therefore replicate not only the tissue’s geometry but also its fixation points to ensure stability. Improper fixation may lead to extrusion, displacement, or abnormal joint mechanics (Stone et al., 1992).

Techniques such as suturing, bone plug fixation, and bioresorbable anchors have been explored, but optimal methods remain unresolved. Additionally, implants must be compatible with arthroscopic surgery, the standard minimally invasive approach for meniscal repair. This imposes constraints on scaffold size, flexibility, and handling characteristics. For example, highly brittle or stiff scaffolds may fracture during insertion, whereas overly soft hydrogels may be difficult to manipulate. Compared to cartilage patches, which can often be secured with adhesives, meniscal scaffolds must accommodate complex loading environments and peripheral anchorage, making surgical integration uniquely challenging (Makris et al., 2011; Stone et al., 1992; Pereira et al., 2019).

## 4 Scaffolds for meniscus tissue engineering

Scaffolds represent a cornerstone of meniscus tissue engineering (TE), providing the structural framework for cellular attachment, proliferation, differentiation, and extracellular matrix (ECM) deposition. An ideal scaffold for meniscus repair must combine biocompatibility, mechanical integrity, biodegradability, and bioactivity, while also recapitulating the anisotropic architecture and zonal heterogeneity of the native tissue. Materials used for meniscus scaffolds can broadly be divided into natural biomaterials, synthetic polymers, and composite constructs. Each category carries distinct advantages and limitations, and recent research has increasingly focused on hybrid approaches that integrate multiple material classes (Lv et al., 2023).

### 4.1 Natural polymer materials

Natural polymers, including collagen, gelatin, hyaluronic acid, chitosan, silk fibroin, alginate, and decellularized extracellular matrix (dECM), are widely investigated for meniscus scaffolds due to their intrinsic bioactivity, cell-friendly surfaces, and ability to promote adhesion and matrix synthesis (Table 2). However, their relatively low mechanical strength, variable degradation rates, and limited structural stability under load often necessitate reinforcement or composite strategies (Chen et al., 2022; Silva et al., 2010). From a comparative and clinical perspective, natural polymer–based scaffolds are particularly advantageous for applications in the avascular inner (white) zone and for partial meniscal defects, where biological induction and matrix remodeling are prioritized over immediate load-bearing capacity. Materials such as collagen, gelatin, hyaluronic acid, and decellularized extracellular matrix provide native biochemical cues that promote cell adhesion, fibrochondrocyte differentiation, and proteoglycan-rich matrix deposition, which are critical for restoring the compressive function of the inner meniscus. However, their limited tensile strength and rapid degradation restrict their standalone use in the vascularized outer (red) zone or in total meniscus replacement, where circumferential hoop stress resistance is essential. Therefore, natural polymers are best suited as bioactive components within composite or layered constructs, rather than as sole structural supports, particularly when long-term mechanical durability is required (Li et al., 2021a; Peng et al., 2022).

**TABLE 2:** Comparison of natural polymer scaffolds in meniscus tissue engineering.

Material category	Representative material	Biological properties	Mechanical strength	Degradation rate	Fabrication/Forming methods	Bioactivity	Key limitations	Representative studies
Collagen-based	Collagen	Excellent biocompatibility, low immunogenicity, RGD-rich	Weak	Moderate (enzymatic)	Freeze-drying, electrospinning, crosslinking	High (promotes ECM synthesis)	Poor suture strength, weak mechanics, batch variability	Pereira et al. (2019), Chiari et al. (2006)
​	Gelatin	Retains RGD motifs, supports cell signaling	Low to moderate	Fast to moderate	Hydrogels, microspheres, GelMA	High (cell-adhesive)	Fast degradation, weak mechanics, requires crosslinking	Tariq et al. (2023), Yue et al. (2015), Van Den Bulcke et al. (2000)
Polysaccharides	Hyaluronic Acid (HA)	Strong hydration, modulates cell behavior via CD44	Weak	Fast	Injectable gels, MeHA photocrosslinking	High (stimulates migration/proliferation)	Poor structural support, fast degradation, needs modification	Burdick and Prestwich (2011), Ansari et al. (2024), Jin et al. (2009)
​	Chitosan	Antibacterial, biodegradable, GAG-like structure	Moderate (if crosslinked)	Slow to moderate	Porous scaffolds, membranes, hydrogels	Moderate (needs blending)	Low adhesion alone, mechanical weakness	Sivanesan et al. (2021), De Sousa Victor et al. (2020), Mao et al. (2003), Jayakumar et al. (2010)
​	Alginate	Easy gelation, low immunogenicity, good for encapsulation	Weak	Fast	Injectable hydrogels, 3D bioink	Low (lacks RGD)	Weak mechanics, no adhesion sites, endotoxin risk	Lee and Mooney (2012), Rastogi and Kandasubramanian (2019), Zhang et al. (2021)
Protein-based	Silk Fibroin	High tensile strength, good elasticity, β-sheet rich	High	Slow	3D printing, freeze-drying, composites	Moderate to high	Brittle under load, difficult fixation, processing sensitivity	Li and Sun (2022), Zhou et al. (2022), Mandal et al. (2011)
Tissue-derived	Decellularized ECM (dECM)	Retains native ECM components, highly biomimetic	Variable	Slow	Hydrogels, powders, composite scaffolds	Very high (strong inductive cues)	Limited source, batch variability, low mechanical strength	Crapo et al. (2011), Neishabouri et al. (2022), Sandmann et al. (2009)

#### 4.1.1 Collagen

Collagen is a natural macromolecular structural protein that is widely present in the connective tissues of the human body. Among them, type I collagen is the most important component in the meniscus. Due to its excellent biocompatibility and low immunogenicity, it has become an ideal candidate material for constructing biomimetic scaffolds (Pereira et al., 2019; Chiari et al., 2006). Collagen can provide abundant cell adhesion sites and promote the synthesis of extracellular matrix, but its mechanical properties are relatively weak, and it needs to be enhanced through structural reinforcement or composite modification (Ranmuthu et al., 2019). Collagen is usually prepared into three-dimensional scaffold structures through processes such as freeze-drying, pneumatic spinning, cross-linking treatment, or by combining with other natural materials. These structures support cell adhesion and proliferation. Some studies have also introduced growth factors onto the surface or constructed microstructures to enhance their cell guidance and tissue integration capabilities (Baek et al., 2022). Dorthé et al. used the pneumatic spinning technique to construct collagen scaffolds with high porosity, which enhanced cell permeability and induced the formation of fibrocartilage-like tissues. The expression of COL1A1 and COL2A1 genes was observed, and the scaffold structure was more similar to the natural meniscus (Dorthé et al., 2022).Baek et al. significantly enhanced cell migration, proliferation and matrix synthesis by covalently binding heparin and growth factors such as TGF-β1, PDGF-BB, and CTGF to the surface of collagen nanofibers, thereby further strengthening their tissue regeneration ability (Baek et al., 2022). Furthermore, Ghodbane et al. combined collagen-hyaluronic acid composite gel with 3D-printed scaffolds, achieving excellent mechanical properties and biological integration in sheep models. The overall performance was close to that of natural tissues (Ghodbane et al., 2019). Although collagen scaffolds have excellent biological activity, they often encounter problems such as insufficient mechanical properties, unstable degradation rate, and poor suture strength in practical applications. Current research mainly focuses on enhancing the structural strength of the scaffolds (such as optimizing the fiber arrangement) and the combined use with functional factors. In the future, further optimization is needed in terms of processability, spatial structure, and biomechanics (Chiari et al., 2006).

#### 4.1.2 Gelatin

Gelatin is a natural protein obtained by hydrolyzing collagen. It retains key bioactive motifs such as Arg-Gly-Asp (RGD), and can effectively mediate cell adhesion and signal transduction. Compared to collagen, gelatin has better solubility and processing flexibility, and is suitable for preparing various forms such as hydrogels, microspheres or films. It also has good biocompatibility and degradability (Tariq et al., 2023). Gelatin is commonly converted into GelMA by reacting with methyl methacrylate (MA). Under light crosslinking conditions, it can rapidly form a hydrogel scaffold, which is suitable for injection and in-situ gelation. In addition, gelatin is also often combined with chitosan, extracellular matrix (ECM), or hyaluronic acid to construct a stable and cell-friendly three-dimensional scaffold, which possesses both biological functions and mechanical support (Yue et al., 2015). Sun et al. evaluated the performance of GelMA in meniscus tissue engineering and found that it was excellent in terms of cell compatibility, but had insufficient mechanical properties (Sun et al., 2018). To improve its structural stability, Zihna et al. covalently combined GelMA with natural ECM to construct a hybrid scaffold with higher strength and thermal stability, providing a design concept for regenerative substitutes (Zihna et al., 2023). Yu et al. further combined the gelatin/chitosan scaffold with the decellularized matrix of the meniscus cells. The experiments proved that this composite scaffold could effectively promote the differentiation of MSCs into the phenotype of meniscus cells, and improve the efficiency of tissue repair (Yu et al., 2019). Although gelatin has excellent biological activity and processing adaptability, its mechanical support capacity for weight-bearing areas is limited. Therefore, cross-linking technology or composite strategies are needed to enhance its strength and stability. Additionally, the degradation rate of gelatin hydrogel structure in the *in vivo* environment is relatively fast. Therefore, the cross-linking density and degradation rhythm need to be further optimized to better match the tissue regeneration process (Yue et al., 2015; Van Den Bulcke et al., 2000).

#### 4.1.3 Hyaluronic acid

Hyaluronic acid is a natural glycosaminoglycan composed of D-glucuronic acid and N-acetyl-D-glucosamine. It is widely present in joint synovial fluid and cartilage. Hyaluronic acid has excellent water retention properties, biocompatibility, and cell signaling regulation capabilities (Burdick and Prestwich, 2011). It can regulate cell proliferation, migration, and inflammatory responses by binding to receptors such as CD44, and is one of the important materials for constructing meniscus tissue engineering scaffolds (Ansari et al., 2024). To enhance its structural stability, hyaluronic acid is often methylacrylated to form methylacrylated hyaluronic acid (MeHA), which is then cross-linked by light to form a stable three-dimensional fiber scaffold. Hyaluronic acid is also commonly combined with natural materials such as collagen and chitosan, or used in combination with MSCs to serve as a delivery carrier for drugs or growth factors. Moreover, due to its hydrophilicity and good cell adhesion properties, hyaluronic acid is also suitable for constructing injectable hydrogel systems (O’shea et al., 2023). Murakami et al. confirmed that hyaluronic acid can activate the PI3K and MAPK pathways, promoting the proliferation of human meniscus cells and inhibiting apoptosis induced by prostaglandin E_2_ (PGE_2_) (Murakami et al., 2019). The high-stiffness MeHA scaffold developed by Song et al. significantly enhanced the matrix deposition and infiltration ability of meniscus cells *in vitro* (Song et al., 2020). Desando et al. conducted experiments on animals and used hyaluronic acid scaffolds loaded with bone marrow mesenchymal stem cells (BMSCs) to treat sheep meniscus defects, achieving significant repair results (Desando et al., 2016). Zellner et al. further demonstrated that in the rabbit model, the use of unpreconditioned BMSCs combined with hyaluronic acid-collagen scaffolds is more conducive to tissue regeneration in the avascular area (Zellner et al., 2013). The degradation rate of hyaluronic acid is fast and its mechanical properties are relatively weak, making it difficult for it to independently fulfill the supporting role of the meniscus in the weight-bearing environment. Its biological effects strongly depend on concentration, cross-linking method, and composite strategy. Future research should focus on its multiple composites with other natural or synthetic materials, as well as its intelligent response performance in controlled release systems (Jin et al., 2009).

#### 4.1.4 Chitosan

Chitosan is a natural polysaccharide obtained by deacetylation of chitin, and it is widely derived from the exoskeletons of crustaceans. Its structure is similar to glycosaminoglycans and it possesses excellent biocompatibility, degradability, and antibacterial properties (Sivanesan et al., 2021).Under physiological conditions, chitosan can slowly degrade into non-toxic products and has certain film-forming properties, making it an important building block for various tissue engineering scaffolds (De Sousa Victor et al., 2020). Due to the insufficient mechanical properties of pure chitosan, in the research, chitosan is often combined with natural materials such as gelatin, collagen or hyaluronic acid to construct porous scaffolds. These scaffolds are usually optimized for pore structure and mechanical strength through processes such as freeze-drying and cross-linking agent treatment (such as using gentiopicroside), and they support cell culture and shaping. Chitosan membranes or hydrogels are also commonly used as cell carriers for meniscus repair (Peng et al., 2022). Sarem et al. designed a gelatin-chitosan porous scaffold and used gentiopicroside as the crosslinking agent, which significantly enhanced its mechanical strength and cell adhesion. At the same time, this scaffold exhibited stress conduction characteristics that were closer to those of the natural meniscus (Sarem et al., 2013a). Subsequently, by adjusting the ratio of gelatin to chitosan and optimizing the freeze-drying process, a three-dimensional scaffold with a pore size of 100–200 μm and a porosity of 80%–90% was prepared, which further enhanced the cell compatibility and structural stability (Sarem et al., 2013b). Moradi et al. conducted experiments in a rabbit meniscus defect model in New Zealand, where they co-cultured polyvinyl alcohol/chitosan scaffolds with adipose-derived mesenchymal stem cells (ADSCs) and chondrocytes. This resulted in the formation of fibrocartilaginous tissue. The results showed that the formed tissue contained abundant glycosaminoglycans (GAGs) and the collagen fibers were arranged in a regular manner, confirming the excellent regenerative potential of this complex (Moradi et al., 2017). The mechanical properties and degradation rate of chitosan scaffolds still cannot fully meet the requirements of the load-bearing environment, and their single use has limited effects. Their performance is highly dependent on the selection of crosslinking agents and the stability of the composite. Future research should continue to optimize its composite formulation, biomimetic design, and nanostructure to enhance its performance in long-term mechanical support and the synergy of biological functions (Mao et al., 2003; Jayakumar et al., 2010).

#### 4.1.5 Silk fibroin

Silk fibroin is derived from the secretions of insects such as silkworms and is a natural structural protein. It is composed of a heavy chain rich in β-sheet structure and a hydrophilic light chain. After removing the silk gum protein, the silk fibroin exhibits excellent biocompatibility, high mechanical strength and excellent elasticity (Li and Sun, 2022). It is a high-quality material for constructing strong and tough tissue scaffolds and is widely used in meniscus tissue engineering (Zhou et al., 2022). Silk fibroin can be processed into various forms of scaffolds through methods such as solution casting, freeze-drying, gas foaming, or 3D printing. In recent years, research has focused on its combined use with components such as ECM, hydrogels, and gelatin, or the combination with cells like MSCs to enhance the biological activity and tissue integration of the scaffolds. Its excellent mechanical properties make it suitable for replacing the partially weight-bearing areas of the meniscus (Ma et al., 2024). Stein et al. evaluated the effect of silk fibroin scaffolds in replacing part of the meniscus in sheep models. The initial mechanical performance was good, but during the medium and long-term follow-up, problems such as increased stiffness and poor integration occurred, suggesting that the stability of these scaffolds still needs to be improved (Stein et al., 2019). Gruchenberg et al. further verified the durability of the silk fibroin scaffold within 6 months of joint loading, but they also observed tears and dislocations at the connection points (Gruchenberg et al., 2015). Ying et al. combined the application of silk fibroin scaffolds with BMSCs in a rabbit model, successfully inducing the formation of meniscus-like tissue, thereby verifying its regenerative ability (Ying et al., 2018). Furthermore, Fritz achieved a significant improvement in the β-pleated structure and compressive modulus by combining silk fibroin with gelatin and ECM and using 3D printing to construct the scaffold, making its mechanical properties closer to those of the natural meniscus (Fritz et al., 2025). Although silk fibroin has good biocompatibility and high mechanical strength, it lacks stability under long-term heavy-load conditions, and the suturing fixation is difficult, which makes it prone to detachment or fracture. Moreover, its structure is susceptible to the influence of processing techniques, and the batch stability needs to be improved. In the future, its integration performance and tissue adaptability can be enhanced through cross-linking reinforcement, biomimetic design, or intelligent composite strategies (Mandal et al., 2011).

#### 4.1.6 Alginates

Alginates are natural polysaccharides extracted from brown algae, possessing excellent gelling properties, hydrophilicity and cell encapsulation characteristics. Their structure is similar to glycosaminoglycans and can form hydrogels by cross-linking with calcium ions under mild conditions (Lee and Mooney, 2012). They are widely used to construct scaffold systems that simulate the microenvironment of soft tissues. Alginate itself has low immunogenicity and is suitable for constructing three-dimensional cell complexes or injectable gels (Rastogi and Kandasubramanian, 2019). In meniscal tissue engineering, alginate is typically used in the form of injectable hydrogels, composite scaffolds, or 3D bioprinting inks. To enhance its biological activity and mechanical properties, it is often mixed with gelatin, carboxymethyl cellulose, ECM, etc., or its degradation rate and crosslinking strength are regulated through oxidation modification. Ultra-purification treatment is also used to reduce the endotoxin content in alginate and improve its biological safety (Zhou et al., 2024). The ultra-pure alginate gel (UPAL) developed by Kim et al. demonstrated excellent regenerative capabilities in the rabbit meniscus defect model, promoting the formation of fibrocartilaginous tissue, and did not induce immune rejection reactions (Kim et al., 2019). The self-crosslinking hydrogel composed of oxidized alginate and gelatin, constructed by Resmi et al., can rapidly gel *in vivo*, significantly enhancing cell adhesion and ECM synthesis capabilities, and promoting tissue repair in pig models (Resmi et al., 2020). Sathish et al. developed a ternary bio-ink containing alginate, gelatin and carboxymethyl cellulose for 3D printing of meniscus scaffolds. *In vitro* experiments showed that it had good cell compatibility, could promote collagen secretion, and improved the mechanical properties of the scaffolds (Sathish et al., 2022). Although alginate has excellent gel properties and embedding capabilities, it naturally lacks cell adhesion sites and has relatively weak mechanical properties, making it difficult to meet the requirements for load-bearing tissue repair. Moreover, the issue of residual endotoxins limits its clinical application. Future research should focus on purification techniques, design of biomimetic complexes, and the introduction of intelligent response functions to enhance its applicability and regulatory capabilities in tissue engineering (Zhang et al., 2021).

#### 4.1.7 Decellularized tissue

Decellularized tissue (dECM) is obtained by physical, chemical or enzymatic methods, during which cells and immunogenic components are removed from the natural tissue while the structural and biochemical composition of the extracellular matrix is retained (Crapo et al., 2011). The naturally preserved components such as collagen, glycosaminoglycans, and elastin in it provide an ideal microenvironment for cell adhesion and function maintenance, making it a highly biomimetic natural scaffold material (Neishabouri et al., 2022). In meniscus tissue engineering, dECM is usually derived from the meniscus tissues of animals such as pigs and rabbits, and is processed into powder, hydrogel or three-dimensional scaffold structures after decellularization. Some studies have combined it with natural polymer materials (such as gelatin, PCL) or synthetic scaffolds to enhance mechanical properties and biological stability, while also considering biological activity. dECM can also serve as a carrier to support the adhesion and directed differentiation of MSCs, thereby improving the repair effect (Xia et al., 2021). Chen et al. obtained porous and hydrophilic porcine dECM scaffolds by using formic acid treatment. After implantation in rats, these scaffolds could promote cell proliferation and cartilage matrix formation, and did not cause significant inflammation (Chen et al., 2015). He et al. further optimized the decellularization process, preserving the collagen and GAG structures, and induced specific regions to undergo re-cellularization in a rabbit model (He et al., 2020). Shadi seeded fibrocartilage cells on the rabbit dECM scaffold and applied dynamic mechanical stimulation. He found that the expression of type I and type II collagen increased, and the tissue structure and mechanical properties improved simultaneously (Shadi et al., 2022). Kara et al. constructed a porcine-derived dECM scaffold through a tripartite decellularization strategy, combined with the implantation of MSCs, achieving significant improvements in cell adhesion, proliferation and ECM synthesis (Kara et al., 2021). A porcine-derived dECM scaffold was constructed through a tri-component decellularization strategy, combined with the implantation of MSCs, achieving significant enhancements in cell adhesion, proliferation, and ECM synthesis. The dECM scaffold exhibits high biomimetic and biological activity, but it also faces issues such as source limitations, batch variations, and complex processing. Additionally, its mechanical properties are usually insufficient and it needs to be combined with high-strength materials for use (Sandmann et al., 2009). In the future, efforts should be focused on standardizing the decellularization process, the controllable degradation characteristics of the materials, and the interface optimization with stem cells, in order to promote its replicability and safety in clinical repair.

### 4.2 Synthetic polymer materials

In the tissue engineering of the meniscus, common polymer materials include polylactic acid (PLA), polyglycolic acid (PGA), Poly (lactic-co-glycolic acid) (PLGA), polycaprolactone (PCL), and polyurethane (PU), etc. These materials are widely used in meniscus tissue engineering due to their excellent biocompatibility, degradability and mechanical properties (Table 3). However, their low biological activity and difficulty in actively inducing cell differentiation are shortcomings that need to be further improved (Makris et al., 2011). In contrast, synthetic polymer scaffolds are more suitable for applications requiring robust initial mechanical support, such as the vascularized outer (red) zone or large, subtotal to total meniscal defects. Materials including PCL, PLGA, PLA, and polyurethane provide superior tensile strength, structural stability, and processability, enabling the fabrication of anatomically precise scaffolds capable of resisting circumferential hoop stresses. These properties are particularly important for maintaining joint biomechanics during the early post-implantation period. However, the inherent bioinert nature of most synthetic polymers limits their ability to actively induce fibrocartilaginous differentiation and extracellular matrix synthesis (Stocco E. et al., 2022; Klarmann et al., 2021). Consequently, their optimal application relies on surface modification, incorporation of bioactive factors, or integration with natural polymers. Taken together, these observations suggest that synthetic polymers alone are insufficient for complete biological regeneration, but are indispensable as mechanical backbones in zone-specific or defect-specific meniscus repair strategies (Zhou et al., 2024; Abbadessa et al., 2021).

**TABLE 3:** Overview of synthetic polymer scaffolds: properties and limitations.

Material	Source and structure	Mechanical strength	Degradability	Processability	Bioactivity	Main limitations	Representative studies
PLA	Aliphatic polyester from renewable sources (e.g., corn, sugarcane)	High	Slow	Excellent (suitable for 3D printing, melt extrusion)	Low (requires modification or blending)	Brittle, acidic degradation products, poor fatigue resistance	Singhvi et al. (2019), Middleton and Tipton (2000)
PGA	Synthetic, high-crystallinity polyester	Moderate	Fast	Good (meshes, fibers, foams)	Low (often combined with bioactive components)	Rapid degradation may outpace tissue formation; limited standalone performance	Gunatillake and Adhikari (2003), Pulapura and Kohn (1992)
PLGA	Copolymer of PLA and PGA with tunable LA/GA ratios	Moderate to high	Tunable (based on ratio)	Good (microspheres, porous scaffolds)	Moderate (supports drug/growth factor delivery)	Acidic degradation by-products, bioactivity depends on functionalization	Makadia and Siegel (2011), Pavot et al. (2014)
PCL	Semi-crystalline aliphatic polyester	High	Very slow	Excellent (3D printable, blendable)	Low (inert surface)	Bioinert, slow degradation may delay tissue integration	Shin et al. (2003), Shcherbakov et al. (2021)
PU	Polyurethane from diisocyanate + polyols	Moderate to high	Moderate to slow (adjustable)	Versatile (injectable, moldable)	Moderate (can carry cells/growth factors)	Long-term stability concerns, potential risk of extrusion or wear in load-bearing sites	Pedersen et al. (2022), Hebda and Pielichowski (2025), Jafari et al. (2017)

#### 4.2.1 Polylactic acid

PLA is a linear polyester formed by polymerization of lactic acid monomers. It is derived from renewable resources such as corn and sugar cane and possesses excellent biocompatibility and degradability. The degradation product of PLA is lactic acid, which can be naturally eliminated through metabolism and has less tissue irritation. It has a high crystallinity and certain rigidity, making it suitable for manufacturing support structures for weight-bearing areas (Singhvi et al., 2019). PLA is usually processed into porous scaffold structures through melt extrusion, solution casting or 3D printing. To address the issues of its high brittleness and insufficient initial mechanical properties, it is often combined with polyhydroxybutyrate (PHB), PCL, hydroxyapatite, etc. Additionally, by surface grafting, plasma treatment or combining with natural polymers (such as collagen, gelatin), its cell adhesion and biological activity can be enhanced (Fredericks et al., 2024). Baek et al. designed an electrospun PLA scaffold and combined human meniscus cells with ECM hydrogel to form a multi-layer composite structure, successfully promoting the formation of meniscus-like tissue (Baek et al., 2015). Gunes et al. constructed a chitosan-collagen hydrogel composite scaffold by combining 3D printing with electrospun nanocellulose, and optimized its biocompatibility and mechanical properties through natural cross-linking agents (Gunes et al., 2022). The PLA/PCL composite scaffold developed by Esposito et al. has shown excellent performance in supporting cell growth and tissue regeneration (Esposito et al., 2013). Although PLA has excellent processing properties and degradability, during its degradation process, acidic intermediate products may be produced, which could affect the local microenvironment; at the same time, it is highly brittle and prone to fatigue failure under complex stress conditions. Future research should focus on regulating its degradation rate, optimizing the composite strategy, and enhancing its biomimetic performance, in order to meet the dual requirements of mechanical and biological functions of the scaffold material for meniscus tissue engineering (Middleton and Tipton, 2000).

#### 4.2.2 Polyglycolic acid

PGA is one of the earliest synthetic polymers used in tissue engineering. It has high crystallinity and rapid degradation characteristics. Its degradation product, glycolic acid, can be metabolized through the tricarboxylic acid cycle and is suitable for tissue repair requiring short-term mechanical support. Due to its poor hydrophilicity and insufficient biological activity, it often needs to be combined with other polymers such as PLA, PCL or natural materials to enhance its biological function and mechanical properties (Gunatillake and Adhikari, 2003). PGA is commonly processed into woven nets, non-woven fabrics and porous scaffolds, and is widely used in sutures and short-term implant materials. Its scaffold forms include nanofibers, three-dimensional printed structures and injectable gels, etc (Xu et al., 2022). The PGA scaffold designed by Otsuki et al. can promote cell growth in the early stage and form the initial cartilaginous tissue (Otsuki et al., 2019). Subsequently, the PGA-coated PLA/PCL copolymer scaffold was developed for the treatment of six patients with irreparable meniscus tears. Clinical follow-up indicated that this scaffold had excellent load-bearing capacity and biological safety (Otsuki et al., 2024). The double-layer composite scaffold (MSS) developed by Ikeda et al. promoted the formation of cartilage-like tissue in the rabbit model, improved the tissue structure, and provided certain protection for the femoral cartilage (Ikeda et al., 2021). However, PGA degrades rapidly and may lose its structural support function before the supporting tissues have fully formed. Its degradation process may also interfere with the quality of the regenerated tissues. Moreover, when used alone, it has weak cell adhesion ability and low biological activity. The improvement directions include combining natural materials to enhance biological activity, designing a structure-function integrated structure, and adopting precise degradation regulation strategies to enhance its application effect in meniscus tissue repair (Gunatillake and Adhikari, 2003; Pulapura and Kohn, 1992).

#### 4.2.3 Poly (lactic-co-glycolic acid)

PLGA is a degradable copolyester formed by the copolymerization and condensation reaction of lactic acid (LA) and glycolic acid (GA). By adjusting the molar ratio of LA to GA, the degradation rate and mechanical properties of PLGA can be flexibly controlled, enabling it to meet the requirements of different stages of tissue repair. PLGA has excellent biocompatibility and processing properties, and is one of the synthetic materials widely studied in meniscus tissue engineering (Makadia and Siegel, 2011). PLGA can be processed into mesh-like, porous or particle composite structures through methods such as solution casting, electrospinning or microsphere encapsulation. Its scaffold forms are diverse, serving both as a structural framework to provide initial mechanical support and as a controlled-release carrier for drugs and biological factors. To enhance its biological activity, PLGA is often combined with platelet-rich plasma (PRP), natural materials or functional molecules (Wyse et al., 2024). Kwak et al. used PLGA mesh scaffolds pre-treated with PRP, which significantly enhanced the adhesion and proliferation ability of fibrocartilage cells, and effectively promoted the repair of meniscus tissue in mice (Kwak et al., 2017). Yoo et al. constructed a xenograft model using porcine chondrocytes and PLGA scaffolds, successfully forming fibrocartilage-like tissue (Yoo et al., 2011). Gu et al. seeded myoblasts onto the PLGA scaffold and observed that they could differentiate into cartilaginous cells expressing type II collagen in the mice (Gu et al., 2013). Li et al. constructed a PLGA microsphere system loaded with Kartogenin (KGN), and embedded it in a PCL scaffold to achieve the sustained release of KGN, inducing the differentiation of human mesenchymal stem cells (hMSCs) into fibrocartilage, and enhancing the biomimetic mechanical properties and tissue induction ability of the scaffold (Li et al., 2021b). However, during the degradation process of PLGA, acidic metabolites may be produced, resulting in a decrease in local pH and affecting cell activity and tissue regeneration. Moreover, its biological activity still relies on the assistance of exogenous factors and is difficult to induce efficient tissue formation alone. Future improvement directions include: optimizing the neutralization mechanism of acidic by-products, developing intelligent controlled-release systems, and combining biomimetic structure design to enhance its biological function adaptability (Makadia and Siegel, 2011; Pavot et al., 2014).

#### 4.2.4 Polycaprolactone

PCL is a synthetic polymer material formed by the ring-opening polymerization of caprolactone. It possesses excellent biocompatibility, slow degradability, and good processability, and is particularly suitable for the preparation of long-lasting load-bearing scaffolds. Its semi-crystalline structure endows it with strong mechanical stability and shaping ability, making it an important candidate material for constructing precise-shaped and mechanically strong meniscus scaffolds (Shin et al., 2003). PCL is particularly suitable for 3D printing and melt molding technologies, enabling the construction of scaffold frameworks with highly biomimetic structures that support cell adhesion, migration, and differentiation. Moreover, PCL is often used in combination with natural polymers (such as silk fibroin, ECM, hydrogels, etc.) to enhance biological activity and cell compatibility (Vyas et al., 2021). Li et al. combined PCL with silk fibroin and fabricated PCL/silk fibroin scaffolds through 3D printing. They verified the excellent tissue regeneration and biomimetic functional recovery effects of these scaffolds in a rabbit meniscus defect model (Li Z. et al., 2020). Zhang et al. implanted PCL scaffolds containing BMSCs in a rabbit model of total meniscus resection. At 12 weeks and 24 weeks, fibrocartilage cells were formed within the scaffolds, expressing type I, type II collagen and proteoglycans. The mechanical properties of the scaffolds with implanted cells were significantly superior to those of the control group without cell implantation (Zhang et al., 2017). The PCL-ligamentous cartilage extracellular matrix (MECM) composite hydrogel scaffold constructed by Chen et al. demonstrated excellent regenerative effects and tissue integration capabilities *in vivo* after seeding fibrocartilage cells (Chen et al., 2019). Although PCL has excellent mechanical strength and processing plasticity, its biodegradation rate is relatively slow, and it may remain in the body even after tissue formation is completed. Moreover, it lacks active sites and requires a combined strategy to enhance its cell responsiveness. Future research should focus on: regulating the degradation rate of PCL (such as copolymerization, surface treatment), enhancing its biological activity functions, and optimizing the printing structure to simulate the stress distribution of the natural meniscus (Shcherbakov et al., 2021).

#### 4.2.5 Polyurethane

PU is a type of synthetic polymer material formed by the polymerization of isocyanates and polyols. It contains amide groups (—NH—COO—) in its structure, which endows it with excellent elasticity, adjustable hardness, and excellent biocompatibility. PU has good processing adaptability and mechanical flexibility, and its degradation rate and mechanical strength can be regulated by different chain segment compositions. It is widely used to construct meniscus tissue engineering scaffolds (Pedersen et al., 2022). PU scaffolds are usually prepared into three-dimensional structures with elastic support through methods such as solution casting, thermoplastic molding or 3D printing. They can withstand the dynamic loads of the knee joint and have a certain degree of shape retention ability. To further enhance their biological activity, PU scaffolds are often combined with MSCs or biological factors to enhance the tissue induction ability (Deng et al., 2021). Bulgheroni et al. evaluated the safety and efficacy of PU scaffolds in the treatment of partial meniscus defects, and found that the MRI results and functional scores of the patients significantly improved 24 months after the surgery (Bulgheroni et al., 2013). Koch et al. further verified the repair potential of the PU scaffolds implanted with MSCs in the rabbit meniscus defect model, observing better angiogenesis and tissue integration (Koch et al., 2018). Condello et al. used aliphatic PU scaffolds to treat patients. During the 36-month follow-up, the knee joint function scores and pain scores of the patients all improved (Condello et al., 2021). Furthermore, studies by Pereira et al. also demonstrated that the combined implantation of PU stents during ACL reconstruction can enhance the stability and regeneration effect of the knee joint (Pereira et al., 2022). Although PU shows promising preclinical effects, the degradation products and the biological inertness after long-term implantation still need further evaluation. Some studies have indicated that PU scaffolds have slight meniscal protrusion and joint cartilage changes in MRI assessment. Future research should focus on: (1) Optimizing the tissue integration ability of the PU structure; (2) Regulating its long-term degradation behavior; (3) Developing functionalized PU scaffolds loaded with stem cells or intelligent release factors to enhance their regenerative effect and clinical safety (Hebda and Pielichowski, 2025; Jafari et al., 2017).

### 4.3 Composite scaffolds

Given the limitations of natural and synthetic materials alone, composite scaffolds have emerged as the most promising strategy for meniscus regeneration. These constructs combine the bioactivity of natural polymers with the mechanical durability of synthetic frameworks (Table 4).

**TABLE 4:** Overview comparison of scaffold materials in meniscus tissue engineering.

Material type	Representative materials	Biocompatibility	Mechanical strength	Degradability	Cost and availability	Processability	Inductive differentiation potential	Main limitations	Representative studies
Natural polymers	Collagen, Gelatin, HA, Chitosan, Silk Fibroin, Alginate	Excellent	Low to Moderate	Fast or Moderate (tunable)	Moderate to High	Good	Strong (can carry growth factors)	Insufficient mechanical strength, batch variability	Chen et al. (2022), Silva et al. (2010)
Synthetic polymers	PLA, PGA, PLGA, PCL, PU	Good	High	Tunable (depends on polymer type)	Moderate	Precise shaping via 3D printing	Weak (requires additional bioactive factors)	Low bioactivity, possible acidic degradation products	Makris et al. (2011), Hebda and Pielichowski (2025), Jafari et al. (2017)
Composite and smart scaffolds	PCL/ECM, PLGA/GelMA, PU/HA/nanoparticles	Excellent	Moderate to High	Precisely tunable	Moderate to High	Excellent (supports 4D/bioprinting)	Strong (programmable release systems)	Complex fabrication, difficult to standardize	Sun et al. (2018), Zhu et al. (2018)

#### 4.3.1 Common combinations

Composite scaffolds that integrate synthetic frameworks with biologically active hydrogels have gained increasing attention in meniscus tissue engineering. For example, polycaprolactone (PCL) combined with decellularized extracellular matrix (dECM) has been shown to provide mechanical stability while simultaneously introducing native biochemical cues; *in vivo* studies demonstrated improved integration and enhanced chondrogenic differentiation compared with PCL alone (Zhu et al., 2018). Similarly, PLGA blended with gelatin methacrylate (GelMA) yields photocrosslinkable constructs with tunable degradation and superior cell adhesion. Under dynamic mechanical stimulation, these scaffolds supported fibrocartilaginous matrix deposition (Sun et al., 2018). Another promising approach involves PCL/PLA frameworks reinforced with alginate hydrogels, which allow simultaneous mechanical support and biochemical microenvironments, facilitating zonal meniscus repair (Xu et al., 2013).

While these hybrid strategies demonstrate significant progress, challenges remain. Variability in dECM composition can affect reproducibility, GelMA-based composites may lack long-term mechanical durability, and alginate provides limited intrinsic bioactivity unless further modified. Nevertheless, the combination of synthetic strength with biological signaling appears to be a key direction for next-generation meniscus scaffolds.

#### 4.3.2 Strategies for optimizing composite scaffolds

Recent studies have sought to refine composite scaffold strategies beyond simple material blending by introducing architectural features that mirror the meniscus’s native heterogeneity. Gradient scaffolds, fabricated through methods such as 3D printing or controlled freeze-casting, introduce spatial variations in stiffness, porosity, and biochemical content, enabling the replication of vascular and mechanical gradients observed between the red and white zones (Luvsannyam et al., 2022). Multiphase composites have been designed with distinct compartments, each optimized for either fibrogenic or chondrogenic differentiation, thereby supporting zone-specific regeneration. Similarly, layered or bilayer scaffolds have been engineered to mimic circumferential fibers on the outer surface while providing compressive resistance internally; such constructs have demonstrated improved tensile properties and matrix organization in preclinical models (Rasheed et al., 2023; Lai and Levenston, 2010).

While these approaches highlight promising design principles, they also face challenges: gradient scaffolds often require complex manufacturing processes, multiphase constructs may suffer from weak interfacial bonding, and layered systems remain to be validated for long-term durability under cyclic joint loading. Nonetheless, these strategies illustrate how composite scaffolds are evolving toward more biomimetic architectures tailored to the unique structural and functional requirements of the meniscus.

#### 4.3.3 Double-layer composite scaffold (MSS)

The term “double-layer composite scaffold (MSS)” refers to a specific strategy wherein one layer is designed for tensile reinforcement (e.g., synthetic aligned fibers) and the other for cell-friendly environments (e.g., hydrogels). Such bilayer constructs aim to reproduce the dual demands of hoop stress resistance and matrix deposition. Recent studies report that double-layer scaffolds seeded with MSCs achieve enhanced fibrocartilaginous tissue formation compared with single-layer constructs (Baek et al., 2015; Kim et al., 2025; Rothrauff et al., 2016).

## 5 Stem cells for meniscus tissue engineering

Cells are indispensable components of tissue engineering strategies, providing the biological activity necessary for matrix deposition, remodeling, and repair. For meniscus regeneration, the choice of cell type and the method of delivery play a pivotal role in determining outcomes. Unlike cartilage tissue engineering, where the primary goal is chondrogenesis, meniscus repair requires a more nuanced approach because the tissue exhibits zonal heterogeneity—fibroblast-like cells dominate the vascularized outer zone, while fibrochondrocytes populate the avascular inner zone. Therefore, engineered constructs must recapitulate both fibrogenic and chondrogenic phenotypes (Donahue et al., 2015).

### 5.1 Mesenchymal stem cells

MSCs are a type of adult stem cells with wide sources and multi-directional differentiation potential. They can differentiate into various cell types such as chondrocytes, adipocytes, and osteoblasts (Li N. et al., 2020). MSCs were first discovered in bone marrow, and subsequently were isolated from various tissues such as adipose tissue, synovium, umbilical cord, amniotic membrane, dental pulp and meniscus. They have a wide range of sources, low immunogenicity, high *in vitro* expansion efficiency, and particularly demonstrate significant advantages in the differentiation of fibrocartilage (Yu et al., 2015; Goldberg et al., 2017).

In meniscus tissue engineering, the role of MSCs is manifested in two core aspects: Firstly, they possess excellent fibrocartilage differentiation ability and can express key cartilage markers such as COL-II and aggrecan (Tsiapalis and O’Driscoll, 2020). Second, it has immunomodulatory functions. It can regulate the local inflammatory microenvironment by secreting cytokines and exosomes, thereby promoting tissue repair and integration (Yang et al., 2023).

At present, the most widely used MSCs are mainly BMSCs, ADSCs and SMSCs. Each of them has its own characteristics in terms of cell activity, differentiation potential and engineering adaptability (Table 5). BMSCs were the first cell type to be applied in meniscus regeneration. They showed good chondrogenic ability and mechanical repair potential in multiple animal experiments, but there was a certain tendency towards chondrocyte hypertrophy. ADSCs have abundant sources, are easy to obtain, and have strong expansion capacity. They have shown good biocompatibility and promoting repair ability in multiple clinical and animal studies, making them suitable for rapid clinical translation. SMSCs are the closest to natural meniscus cells in terms of maintaining fibrocartilaginous phenotype, extracellular matrix synthesis and structural simulation. They are one of the most promising seed cells at present (Elkhenany et al., 2021; Bian et al., 2022; Rady et al., 2020).

**TABLE 5:** Comparison of different sources of MSCs for meniscus tissue engineering.

Feature	BMSCs (bone marrow MSCs)	ADSCs (adipose-derived MSCs)	SMSCs (synovium-derived MSCs)
Ease of harvest	Moderate	Easy	Easy
Fibrochondrogenic potential	High	Moderate	Very High
Proliferation ability	Moderate	High	High
Immunogenicity	Low	Low	Low
Suitability for tissue engineering	✔	✔	✔ (Most promising)
Main limitation	Risk of hypertrophic differentiation	Unstable phenotype commitment	Limited clinical experience
Representative studies	Elkhenany et al. (2021), Yan et al. (2025)	Zheng et al. (2023), Li et al. (2018), Lee et al. (2013)	Li et al. (2020b), Ding et al. (2022), Nakamura et al. (2024)

#### 5.1.1 Bone marrow mesenchymal stem cells (BMSCs)

BMSCs is the earliest type of mesenchymal stem cells applied in meniscus tissue engineering. It was initially isolated from bone marrow. It has excellent fibrocartilage differentiation ability, biocompatibility and low immunogenicity. In several studies, BMSCs demonstrated strong expression capabilities of COL-II and proteoglycan (Aggrecan), and are one of the key seed cells for meniscus regeneration. However, the acquisition of BMSCs is relatively invasive, and the *in vitro* expansion efficiency is moderate, with a tendency towards cartilage hypertrophy (Elkhenany et al., 2021). BMSCs are usually induced and cultured *in vitro* and then combined with biomimetic scaffolds for meniscus repair. The scaffold materials include collagen, PLGA, PCL, etc. Researchers have also explored methods such as adjusting oxygen tension, biological factors (such as TGF-β), and mechanical strain (such as stretching stimulation) to induce their differentiation into fibrocartilage phenotype (Li et al., 2021a). Elkhenany et al. conducted experiments by inoculating BMSCs onto collagen scaffolds and culturing them under intermittent hypoxic tension. The results showed that the extracellular matrix synthesis ability and mechanical properties of these cells were significantly superior to those of the control group. However, there was an increase in the expression of type X collagen (COL10), suggesting a tendency towards hypertrophy (Elkhenany et al., 2021). The experiment conducted by Su et al. revealed that a 10% radial tensile strain was the most conducive to inducing BMSCs to differentiate into fibrocartilage, while a 15% strain might trigger the expression of α-smooth muscle actin (α-SMA), resulting in matrix contraction and structural instability (Su et al., 2020). Perea et al. further confirmed that the serum-free culture system combined with TGF-β and dexamethasone could enhance the efficiency of matrix deposition and the biomechanical properties (Hidalgo et al., 2020). BMSCs are promising for meniscus tissue engineering because of their capacity to produce extracellular matrix and differentiate toward fibrocartilage. However, several limitations are well documented. In comparative *in vitro* studies, when seeded on aligned electrospun nanofiber scaffolds without additional growth factor stimuli, BMSCs exhibit fibroblastic—not fully chondrogenic—phenotypes, while chondrocytes maintain superior expression of cartilage-associated genes and matrix components, indicating strong dependence on inductive cues (Zheng et al., 2023). Clinically, BMSCs are harvested via bone marrow aspiration, which is invasive and may cause donor site pain and risk of infection—a barrier acknowledged in numerous reviews (Li et al., 2021a). To address these challenges, recently developed smart scaffolds have incorporated shape-memory skeletons and adhesives to improve implant stability, reduce trauma from surgical delivery, and enhance chondroprotective outcomes in rabbit models. While direct evidence on suppression of hypertrophy (e.g., COL10) *in vivo* remains limited, future work should emphasize optimizing the culture microenvironment (e.g., hypoxia, addition of anti-hypertrophic growth factors), careful modulation of scaffold biophysical cues, and integration of controlled release systems to stabilize the fibrocartilaginous phenotype of BMSCs under weight-bearing conditions (Yan et al., 2025).

#### 5.1.2 Adipose-derived mesenchymal stem cells (ADSCs)

ADSCs originate from subcutaneous adipose tissue. The acquisition method is minimally invasive, has a rich source, and has strong expansion capacity. It is a type of seed cell with great clinical application prospects. Although its potential for fibrocartilage differentiation is slightly lower than that of BMSCs, it has excellent biocompatibility and is suitable for application in injection or minimally invasive treatment strategies (Li et al., 2018). ADSCs are commonly used in injectable cell therapies, or are combined with three-dimensional scaffolds (such as collagen sponges, layered materials) and implanted into the defect area. To enhance their chondrogenic differentiation ability, studies usually induce and cultivate the cells with biological factors such as TGF-β and IGF. Techniques such as cell layer-embedding and cell cluster embedding are also applied to improve cell density and spatial organization structure (Moncada-Saucedo et al., 2019). The clinical research conducted by Pak et al. demonstrated that after autologous ADSCs were directly injected into the area of meniscus tear, the pain score of the patients decreased, and MRI showed that the damaged area partially closed (Pak et al., 2014). Toratani et al. conducted an animal experiment in which they implanted three-dimensional cell clusters of ADSCs into the defect area. The results showed that the expressions of proteoglycans and COL-I significantly increased (Toratani et al., 2017). Takata and the Kimura team used cell layer sheets to enhance the mechanical properties of tissues (Takata et al., 2020; Kimura et al., 2023). Itose et al. found in their study of the use of unamplified ADRCs in combination with collagen sponges that this strategy was helpful in increasing COL-II expression and biomechanical properties (Itose et al., 2022). Although ADSCs demonstrate excellent ease of acquisition and proliferation rate, their fibrocartilaginous phenotype is unstable and is prone to phenotypic drift due to the influence of the microenvironment. Moreover, their terminal differentiation ability is limited, and there are still risks of chondrocyte hypertrophy and non-target differentiation. Future research can consider enhancing the efficiency of fibrocartilaginous differentiation through gene modification, intelligent scaffold induction, and multi-factor co-stimulation, and exploring its potential in personalized treatment (Zheng et al., 2023; Lee et al., 2013).

#### 5.1.3 Synovial mesenchymal stem cells (SMSCs)

SMSCs originate from the synovial tissue of joints and are relatively easy to obtain. They possess extremely strong chondrogenic differentiation ability, high proliferative potential and low immunogenicity. Compared with BMSCs and ADSCs, SMSCs are more similar to natural meniscus cells in terms of maintaining fibrocartilaginous phenotype, synthesis of extracellular matrix (ECM) and structural mimicry. They are currently regarded as one of the most promising types of seed cells (Ding et al., 2022). SMSCs are usually combined with natural or composite scaffolds (such as decellularized meniscus scaffolds, electrospun scaffolds, etc.) and induced to differentiate by adding chondrogenic factors (such as TGF β1, IGF 1, etc.). In addition, when combined with the biaxially arranged biomimetic scaffold structure or dynamic mechanical stimulation, it can further promote their directional differentiation and functional tissue formation (Shimomura et al., 2019). Kondo et al. implanted autologous SMSCs in an elderly primate model and observed that the regenerated tissue was morphologically and compositionally similar to the original meniscus (Kondo et al., 2017). Ozeki et al. demonstrated in a model of vascular-deficient area in pigs that SMSCs significantly promoted the formation and integration of the regenerative tissue structure (Ozeki et al., 2021). Liang et al. implanted SMSCs into the decellularized meniscus matrix scaffold and combined with TGF β1 and IGF 1 for induction culture. This significantly increased the expression of fibrocartilage-specific markers and enhanced the synthesis of GAG and type II collagen (Liang et al., 2018). Furthermore, Shimomura et al. designed an electrospun nanoscale scaffold with a circular fiber arrangement, successfully promoting the directional adhesion and differentiation of SMSCs, and enhancing the mechanical properties and tissue integration of the regenerated tissue (Shimomura et al., 2019). Although SMSCs possess excellent biological properties, their clinical research is still insufficient, and standardized extraction and cultivation procedures still need to be established. Moreover, SMSCs from different patient sources may have phenotypic differences and functional fluctuations. In the future, efforts should be made to strengthen the study of the mechanisms underlying their biological properties, and develop exclusive and efficient induction systems and intelligent scaffolds for SMSCs to enhance their clinical controllability and applicability (Li N. et al., 2020; Nakamura et al., 2024).

### 5.2 Induced pluripotent stem cells (iPSCs)

iPSCs are pluripotent cells derived from somatic cells (such as skin or blood cells) through reprogramming technology. They possess similar self-renewal and tri-lineage differentiation potential to embryonic stem cells. They have a wide range of sources, high individuality, and can avoid immune rejection issues, making them a promising new cell source in regenerative medicine (Hirschi et al., 2014). The application of iPSCs in meniscus tissue engineering mainly relies on their ability to be induced into fibrocartilage-like cells. The current commonly used methods include embryoid body induction method and gene editing-assisted induction (such as using factors like TGF-β3, BMP-2, etc.), and some studies also combine scaffold carrier systems and directed release strategies for combined cultivation. In addition, gene editing technologies such as CRISPR/Cas9 are used to improve the differentiation purity and functional specificity of iPSCs (Adkar et al., 2019). Costa et al. and Rim et al. have confirmed that iPSCs can successfully be induced to form fibrocartilaginous cells expressing SOX9, COL2A1, and Aggrecan in an environment containing factors such as TGF β1, TGF β3, and BMP 2 (Costa et al., 2017; Rim et al., 2020). Brunger et al. knocked out the inflammatory-related gene IL1R1, which improved the tolerance of miPSCs to the inflammatory microenvironment and enhanced their ability to generate cartilage matrix (Brunger et al., 2017). Dicks et al. established the COL2A1-GFP reporting system and, in combination with CRISPR screening, obtained the expression of surface markers such as CD146^+^/PDGFRβ^+^ of chondroprogenitor cells, significantly improving the purity and consistency of chondrocytes (Dicks et al., 2020). Although iPSCs possess unlimited proliferation and differentiation potential, there are still significant challenges in terms of induction efficiency, phenotypic consistency, and safety. Firstly, the differentiation process is complex, which may lead to heterogeneity and non-target differentiation. Secondly, reprogramming and genetic manipulation carry potential oncogenic risks, restricting their clinical application. Moreover, the standardized preparation process and long-term *in vivo* evaluation data are still insufficient. Future research should focus on: (1) Developing non-integrated reprogramming systems to reduce genomic instability; (2) Designing efficient and stable induction differentiation systems; (3) Combining intelligent scaffolds and targeted release platforms to achieve safe, controllable, and individualized clinical application pathways (Zhong et al., 2022; Lemmens et al., 2023; Haridhasapavalan et al., 2019).

### 5.3 Cell seeding strategies

Beyond the choice of cell source, the strategy used for delivering cells into scaffolds or defect sites plays a decisive role in determining regenerative outcomes. In the literature, the term “*seed cells*” has occasionally been used; however, this phrasing is unconventional. More accurate terminology includes “*cell seeding*” (the process) or “*cell-seeded scaffolds*” (the product), which better reflect accepted usage in tissue engineering research (Dorthé et al., 2022; Vacanti and Langer, 1999).

#### 5.3.1 Static seeding

Cells are pipetted or incubated onto scaffold surfaces, relying on passive adhesion and infiltration. While simple and cost-effective, static seeding often results in uneven distribution and poor penetration into porous scaffolds (Chen et al., 2025; Shi et al., 2022).

#### 5.3.2 Dynamic seeding

**FIGURE 4:**
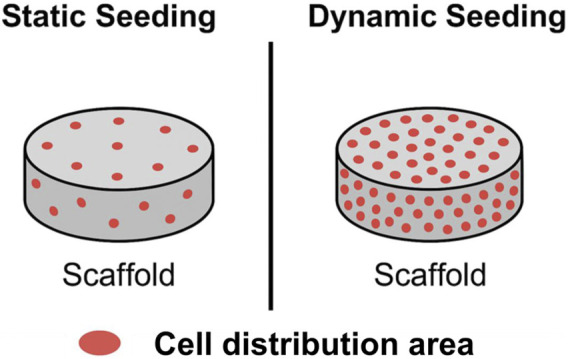
This diagram illustrates two common methods for seeding cells onto scaffolds in meniscus tissue engineering. It compares static seeding, where cells are placed on scaffold surfaces but may suffer from poor penetration and uneven distribution, dynamic seeding, which utilizes fluid flow to enhance cell distribution and nutrient transport.

Dynamic methods use fluid flow or mechanical agitation to drive cells into scaffold pores, enhancing distribution and nutrient transport. Perfusion bioreactors not only improve seeding efficiency but also allow the application of shear stress, which promotes chondrogenic differentiation (Kock et al., 2014; Engel et al., 2021) (Figure 4).

#### 5.3.3 3D bioprinting and co-printing approaches

Recent advances in 3D printing allow for precise spatial placement of cells within bioinks, enabling constructs that mimic zonal organization of the meniscus. Co-printing stem cells with hydrogels (e.g., GelMA, alginate) into synthetic frameworks (e.g., PCL) yields hybrid structures with both mechanical support and biological functionality (Chae et al., 2021; Yang et al., 2022) (Table 6).

**TABLE 6:** Cell seeding strategies comparison table.

Method	Advantages	Limitations	Representative studies
Static seeding	Simple and cost-effective; widely used; minimal equipment required	Often results in uneven cell distribution; poor infiltration into scaffold interior	Chen et al. (2025), Shi et al. (2022)
Dynamic seeding	Improves cell penetration and distribution; enhances nutrient and oxygen transport; mimics physiological flow conditions	Requires specialized bioreactors; more complex and costly; shear stress may affect cell viability if not controlled	Kock et al. (2014), Engel et al. (2021)
3D bioprinting	Allows precise spatial control of cells and biomaterials; enables zonal organization; integrates multiple cell types and materials	High technical demand; bioink limitations; may compromise cell viability during printing; scalability remains challenging	Chae et al. (2021), Yang et al. (2022)

#### 5.3.4 Injection-based delivery

In some strategies, stem cells are directly injected into defects, often with hydrogels as carriers. Although minimally invasive, this approach faces persistent challenges with cell retention and integration. These limitations underscore the broader need to evaluate cell delivery methods within the context of meniscus-specific biology (Jacob et al., 2019; Li et al., 2023).

Cell sources and seeding strategies play an essential role in meniscus tissue engineering. BMSCs, ADSCs, and SMSCs each offer distinct advantages tailored to specific meniscal zones, while iPSCs represent a powerful but still experimental platform requiring careful safety validation. Advances in seeding technologies, particularly dynamic perfusion and 3D bioprinting, are expanding the ability to recreate the meniscus’s zonal heterogeneity. A nuanced combination of cell type and delivery strategy will be necessary to overcome the unique biological challenges of meniscal repair (Deng et al., 2021; Zheng et al., 2023; Du et al., 2023).

## 6 Emerging technologies and future perspectives

Meniscus tissue engineering has advanced considerably over the past 2 decades, yet challenges such as mechanical mismatch, limited vascularization, and incomplete integration remain unsolved (Lorenz et al., 2022). To address these barriers, researchers have increasingly turned to novel technologies that merge engineering, materials science, and computational design. Among the most promising are 3D and 4D printing techniques, responsive biomaterials with targeted drug delivery capabilities, dynamic culture systems that mimic physiological loading, and artificial intelligence (AI)-assisted scaffold design (Arias-Peregrino et al., 2025). Together, these approaches hold the potential to transform meniscus tissue engineering from a laboratory concept into a clinically viable solution.

### 6.1 3D and 4D printing technologies

Additive manufacturing has revolutionized scaffold fabrication by enabling precise control over geometry, porosity, and fiber orientation. For the meniscus, 3D printing allows the production of patient-specific implants tailored to MRI or CT imaging data, ensuring accurate anatomical fit (Shadi et al., 2022; Loverde et al., 2023; Szojka et al., 2021a).

**FIGURE 5:**
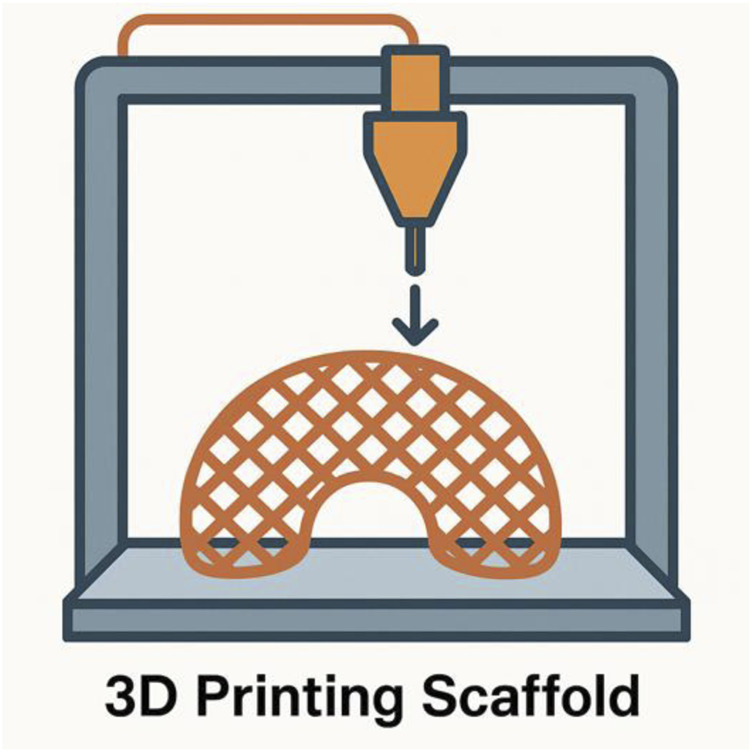
3D printing technology.

Additive manufacturing has enabled the fabrication of meniscus-specific scaffolds with controlled architecture and composition. Polycaprolactone (PCL) scaffolds with aligned fibers, for example, have been 3D printed to replicate circumferential collagen organization, resulting in improved tensile properties and hoop stress resistance compared with isotropic constructs (Guo et al., 2021). However, while mechanically robust, PCL lacks inherent bioactivity. To address this limitation, hybrid scaffolds combining PCL frameworks with hydrogels such as GelMA or alginate have been reported, demonstrating more uniform cellular distribution and enhanced fibrocartilaginous matrix formation under mechanical stimulation (Chen et al., 2019; El Kommos et al., 2025). Similarly, the use of decellularized extracellular matrix (dECM) bioinks has introduced scaffolds enriched with native biochemical cues, supporting both proliferation and chondrogenic differentiation of seeded cells (Golebiowska et al., 2025). Yet, variability in dECM sources and potential immunogenicity remain concerns for clinical translation (Figure 5).

Beyond 3D printing, 4D printing approaches have been proposed, wherein scaffolds are programmed to undergo time-dependent shape changes or respond to external stimuli. Hydrogel-based constructs capable of swelling or bending under hydration have been designed to mimic the adaptive deformation of the meniscus during joint loading (Liu et al., 2024). Although these concepts remain at an early stage, they highlight the potential for implants that dynamically adapt to *in vivo* mechanical environments (Patrawalla et al., 2024).

Collectively, additive manufacturing offers unprecedented precision and patient-specific customization, but challenges persist in scaling production, ensuring sterility, and validating long-term biomechanical durability. Addressing these issues will be essential before 3D/4D printing can be widely applied in clinical meniscus repair (Wang Z. et al., 2025).

### 6.2 Smart biomaterials and drug delivery systems

Traditional scaffolds often passively support cell growth, but next-generation designs incorporate responsive biomaterials capable of adapting to environmental cues or delivering therapeutic molecules in a controlled manner (Gelmi and Schutt, 2021).

#### 6.2.1 Stimuli-responsive hydrogels

**FIGURE 6:**
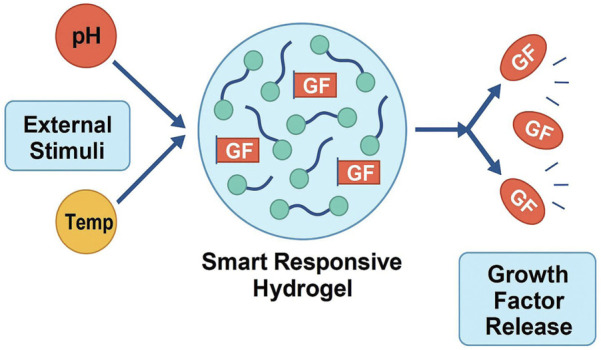
This diagram illustrates the function of stimuli-responsive hydrogels in meniscus tissue engineering. The hydrogels are designed to release bioactive factors, such as TGF-β3 and IGF-1, in response to environmental stimuli, such as pH, temperature, or enzymatic activity. This controlled release supports the chondrogenic differentiation of stem cells into fibrocartilage-like phenotypes, essential for meniscus repair.

Hydrogels that respond to pH, temperature, or enzymatic activity can release bioactive factors in synchrony with cellular needs (Figure 6). For meniscus repair, thermoresponsive hydrogels have been developed that gel at body temperature, allowing minimally invasive injection into defects followed by *in situ* scaffold formation. Similarly, enzyme-sensitive hydrogels degrade in response to matrix metalloproteinases, releasing growth factors only in remodeling environments (Qu et al., 2020; Sobczak, 2022).

#### 6.2.2 Growth factor delivery

Delivery of factors such as TGF-β3, IGF-1, and connective tissue growth factor (CTGF) has shown promise in directing stem cell differentiation toward fibrochondrocyte-like phenotypes (Moioli et al., 2006). Controlled release strategies, such as encapsulation in microspheres or tethering to hydrogel matrices, help maintain local concentrations over clinically relevant timeframes. For instance, composite scaffolds integrating PU frameworks with microsphere-loaded hydrogels have demonstrated sustained TGF-β3 release, resulting in enhanced type I and II collagen deposition (Lin et al., 2021).

#### 6.2.3 Anti-inflammatory and pro-angiogenic agents

Smart scaffolds may also be designed to release anti-inflammatory molecules (e.g., dexamethasone) to counteract the catabolic environment of injured joints or pro-angiogenic factors (e.g., VEGF) in a spatially controlled fashion to encourage vascularization in the red zone while leaving the white zone avascular (Pisani et al., 2023; Freeman et al., 2021).

By coupling mechanical reinforcement with biochemical delivery, smart biomaterials offer a path toward multifunctional scaffolds capable of addressing multiple regenerative challenges simultaneously (Su et al., 2020; Sun et al., 2023).

### 6.3 Dynamic culture systems and biomechanical stimulation

The meniscus functions in a mechanically demanding environment, experiencing cyclic compression, tension, and shear during joint motion. Static culture systems fail to replicate these conditions, often resulting in constructs with suboptimal matrix composition and organization. Dynamic culture platforms and bioreactors aim to overcome this limitation by providing physiologically relevant mechanical stimulation (Aufderheide and Athanasiou, 2004).

#### 6.3.1 Bioreactor systems

Perfusion bioreactors improve nutrient and oxygen transport while applying controlled shear stress, which has been shown to enhance GAG deposition and chondrogenic differentiation of MSCs seeded in meniscus scaffolds (Liu et al., 2012).

Compression bioreactors simulate axial joint loading, encouraging fibrocartilaginous ECM production and aligning collagen fibers.

Rotating wall vessel bioreactors create microgravity-like conditions that promote uniform cell distribution and tissue maturation (Jaasma et al., 2008).

#### 6.3.2 Mechanical stimulation strategies

Biomechanical stimulation has emerged as a critical factor in guiding zone-specific differentiation within engineered meniscus constructs. Cyclic tensile strain applied to scaffolds with aligned fibers has been reported to promote fibroblast-like differentiation and enhance type I collagen deposition, favoring regeneration of the tensile outer zone (Yang et al., 2025). In contrast, dynamic compressive loading stimulates chondrogenic differentiation and glycosaminoglycan accumulation, supporting fibrocartilaginous matrix formation characteristic of the inner zone (Chen et al., 2018). More recently, combined loading protocols that integrate both tensile and compressive forces have been employed to better replicate *in vivo* joint mechanics, resulting in anisotropic extracellular matrix (ECM) deposition resembling the native meniscus (Huey and Athanasiou, 2011).

While such bioreactor-based strategies have improved preclinical outcomes, they also serve as valuable platforms for elucidating mechanobiological pathways in meniscal cells. Nonetheless, scaling these systems to produce clinically relevant grafts remains a considerable engineering and logistical challenge, underscoring the need for streamlined, reproducible bioreactor designs (Radisic et al., 2008; Petri et al., 2012).

### 6.4 AI-assisted scaffold design and personalized medicine

Artificial intelligence (AI) and computational modeling are increasingly being integrated into biomaterials research to accelerate scaffold design and optimize clinical outcomes (Madika et al., 2025) (Table 7).

**TABLE 7:** Emerging technologies and applications in meniscus TE.

Techology	Key features	Current meniscus applications	Future challenges	Representative studies
3D/4D printing	Additive manufacturing with precise control over geometry, porosity, and fiber alignment; 4D printing allows time-dependent shape transformation or responsiveness	PCL scaffolds with aligned fibers for hoop stress resistance; hybrid constructs with hydrogels; dECM-based bioinks for cell-laden printing	Scaling for clinical use; ensuring sterility; validating long-term durability *in vivo*; regulatory approval	Chen et al. (2019), Guo et al. (2021), El Kommos et al. (2025), Golebiowska et al. (2025)
Smart hydrogels	Stimuli-responsive hydrogels (pH, temperature, enzymes); can undergo gelation *in situ* or release biomolecules in response to local environment	Thermoresponsive or enzyme-sensitive gels used to encapsulate cells or release TGF-β and IGF-1 *in situ*	Ensuring mechanical robustness; tuning degradation to match healing; avoiding immune reactions; reproducibility of formulations	Qu et al., 2020; Sobczak (2022)
Drug delivery systems	Controlled release of growth factors, anti-inflammatory agents, or angiogenic molecules; may use microspheres, nanoparticles, or tethered	Composite scaffolds delivering TGF-β3, IGF-1, or VEGF to promote fibrocartilaginous matrix deposition and vascularization in outer zone	Achieving spatiotemporal control of release; preventing burst release; maintaining bioactivity; regulatory complexity	Lin et al. (2021)
AI-assisted scaffold design	Uses computational modeling and machine learning to optimize scaffold design, predict performance, and tailor implants to patient-specific anatomy and biomechanics	Preclinical stage; early studies integrating imaging data with finite element analysis to design patient-specific meniscus implants	Need for large validated datasets; integration with clinical workflows; regulatory approval; validation of AI-generated designs *in vivo*	Pais et al. (2023), Omigbodun and Oladapo (2025)

#### 6.4.1 Design optimization

Machine learning algorithms can analyze large datasets of scaffold compositions, microarchitectures, and biomechanical outcomes to predict the optimal parameters for meniscus repair. Generative design approaches allow for automated creation of scaffold architectures tailored to specific mechanical and biological targets (Pais et al., 2023; Omigbodun and Oladapo, 2025).

#### 6.4.2 Patient-specific modeling

By integrating imaging data (MRI, CT) with finite element modeling, AI systems can simulate patient-specific knee biomechanics, enabling the design of custom scaffolds that reproduce load distribution and deformation patterns unique to each individual. This approach holds promise for personalized meniscus implants that maximize integration and minimize extrusion (Ma et al., 2025; Esrafilian et al., 2025).

#### 6.4.3 Predictive analytics for outcomes

AI models may also be used to forecast long-term outcomes based on patient demographics, injury type, scaffold design, and rehabilitation protocols, guiding clinical decision-making (Jang et al., 2024; Buscarini et al., 2025).

Although AI-assisted scaffold design remains largely conceptual at present, its integration with 3D printing, computational biomechanics, and patient imaging heralds a new era of personalized regenerative medicine. The synergy between advanced manufacturing and intelligent design has the potential to accelerate translation while reducing trial-and-error experimentation (Szojka et al., 2021b; Bian et al., 2025).

## 7 Limitations of tissue-engineered meniscus

Despite significant advances, tissue-engineered meniscus constructs remain associated with several limitations that hinder clinical translation. Achieving sufficient mechanical strength and fatigue resistance under repetitive joint loading remains a major challenge, particularly as scaffolds undergo degradation and remodeling.

In addition, consistent integration with native meniscal tissue, vascular interfaces, and horn attachments has not been reliably achieved. Manufacturing complexity, batch-to-batch variability, and the lack of standardized evaluation protocols further complicate regulatory approval and large-scale clinical adoption. These limitations highlight the need for cautious interpretation of preclinical success and underscore the importance of long-term *in vivo* and clinical studies.

## 8 Conclusion

Meniscus tissue engineering has made substantial progress over the past decade, driven by advances in biomaterial science, stem cell biology, and biofabrication technologies. A wide range of scaffold materials—including natural polymers such as collagen, alginate, and silk fibroin, as well as synthetic polymers such as PGA, PLA, PLGA, and polyurethane—have provided essential platforms for supporting cellular adhesion, proliferation, and differentiation. In particular, composite scaffolds that integrate the bioactivity of natural materials with the mechanical stability of synthetic polymers have emerged as a promising strategy, enabling the fabrication of multiphase, gradient, or layered constructs that more closely recapitulate the anisotropic architecture of the native meniscus. Parallel progress in stem cell–based approaches, including the use of bone marrow–, adipose-derived, and synovial mesenchymal stem cells, has expanded the regenerative toolkit, while advances in cell delivery methods—such as dynamic seeding, perfusion systems, and 3D bioprinting—have improved cell distribution, viability, and scaffold–cell integration.

Despite these encouraging developments, significant challenges continue to limit the clinical translation of meniscus tissue engineering. Replicating the meniscus’s unique zonal vascularization remains difficult, as strategies must promote revascularization of the outer red zone without compromising the avascular inner white zone. Similarly, reconstructing the circumferential collagen fiber architecture and radial tie fibers essential for hoop stress resistance remains a major hurdle, as many current scaffolds lack sufficient tensile anisotropy and long-term durability under cyclic loading. Mechanical mismatch, incomplete integration with native tissue, and degradation profiles that are poorly synchronized with tissue remodeling further contribute to inconsistent outcomes in preclinical and early clinical studies. These challenges highlight that successful meniscus regeneration requires not only biological viability, but also durable biomechanical performance under physiological joint loading.

**FIGURE 7:**
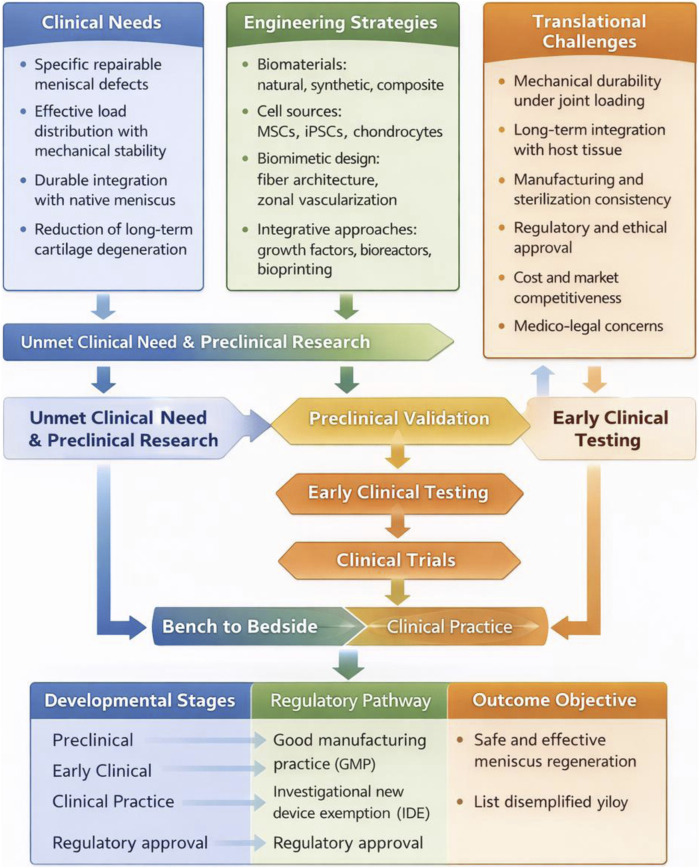
Illustrates a translational roadmap for meniscus tissue engineering, integrating clinical needs, engineering strategies, and key translational challenges along the bench-to-bedside pathway. Clinical requirements, including durable load distribution, stable integration with native tissue, and prevention of long-term cartilage degeneration, define the unmet therapeutic demands. Engineering strategies—encompassing biomaterial selection, cell sources, and biomimetic scaffold design—aim to address these needs through preclinical validation and early clinical testing. Major translational challenges, such as mechanical durability under joint loading, long-term integration, manufacturing consistency, regulatory approval, and medicolegal considerations, critically influence the progression toward clinical trials and routine clinical practice. This schematic highlights the interdependence of biological, mechanical, and regulatory factors required for the development of clinically reliable tissue-engineered meniscus constructs.

From a clinical perspective, the translation of tissue-engineered meniscus constructs must be approached with caution and responsibility. At present, only a limited number of meniscal implants are available for clinical use, and reported complications—including implant extrusion, mechanical failure, and progression of cartilage degeneration—underscore the importance of careful patient selection. For clinicians, the decision to implant synthetic or tissue-engineered meniscal substitutes carries not only therapeutic promise but also medicolegal responsibility, particularly in the absence of definitive long-term outcome data. Future progress will therefore depend on rigorous, well-designed clinical trials, continued refinement of biomimetic scaffold design, and transparent communication among researchers, clinicians, industry partners, and regulatory authorities. Only through evidence-based integration can tissue-engineered meniscus technologies transition from experimental innovation to reliable and responsible clinical practice.The interdependent biological, mechanical, and translational factors governing the clinical viability of meniscus tissue engineering are summarized in Figure 7, which outlines a roadmap from unmet clinical needs to responsible clinical application.
